# Network pharmacology and molecular modelling analysis of *Arctium lappa* phytochemicals in valproic acid–induced hepatotoxicity and pancreatitis

**DOI:** 10.3389/fbinf.2026.1870490

**Published:** 2026-07-20

**Authors:** Mukul Shyam, Sabina Evan Prince

**Affiliations:** School of Bio Sciences and Technology, VIT University, Vellore, India

**Keywords:** hepatotoxicity, molecular dynamics simulation, network pharmacology, pancreatitis, valproic acid

## Abstract

**Background:**

Valproic acid (VPA) is an antiepileptic drug commonly employed to treat epilepsy. Despite this, the clinical application of VPA is often hindered by adverse reactions. This study aims to investigate the therapeutic potential of phytochemicals obtained from *Arctium lappa* to mitigate VPA-induced toxicity and to determine the mechanisms involved.

**Methods:**

Network pharmacology and molecular modelling techniques were employed. HR-LCMS-QTOF was conducted to identify the phytoconstituents. ADMET and toxicity screening were then performed. Ligand-based prediction and toxicity gene analysis were employed to determine the potential targets. Protein-protein interaction analysis and function enrichment analysis were conducted to understand the potential mechanisms. Molecular docking and molecular dynamics studies were conducted to determine the protein-ligand interaction potential.

**Results:**

A total of 111 metabolites were identified, out of which 22 compounds passed the ADMET and toxicity screening. Using ligand-based prediction and toxicity analysis, 18 potential target genes were identified. These include MAPK8, NLRP3, TNF-α, and MCL-1. These are involved in various functions, including inflammation, apoptosis, and metabolic regulation. Molecular docking analysis revealed high protein-ligand interaction potential. This was evident by the high binding affinity of the phytoconstituents to the potential targets. Among all the compounds, 4-Hydroxy-3-methoxy-2,10-bisaboladien-9-one showed the highest potential. Molecular dynamics analysis revealed high protein-ligand complex stability, especially with MCL-1 and NLRP3. Function enrichment and Metascape analysis revealed the involvement of the potential targets in oxidative stress, inflammation, and xenobiotic metabolism.

**Conclusion:**

The results suggest that phytoconstituents from *A. lappa* have the potential to modulate key molecules involved in VPA-induced toxicity.

## 1 Background

Epilepsy is a chronic neurological disorder characterized by recurrent, unprovoked seizures resulting from abnormal neuronal excitability and synchronization in the brain. It affects approximately 50 million people worldwide, making it one of the most common neurological conditions, with a higher prevalence in low- and middle-income countries (Thijs et al., 2019). The disorder imposes a significant socioeconomic burden due to its chronic nature, associated comorbidities, and impact on quality of life. Despite advances in pharmacotherapy, nearly one-third of patients remain drug-resistant, highlighting the need for safer and more effective therapeutic strategies (Kwan et al., 2010). Valproic acid (VPA) is a broad-spectrum antiepileptic drug widely used in the management of epilepsy, bipolar disorder, and migraine prophylaxis. Its mechanism of action is multifaceted, involving enhancement of γ-aminobutyric acid (GABA)-mediated inhibitory neurotransmission, modulation of voltage-gated sodium and calcium channels, and inhibition of histone deacetylases (HDACs), which contributes to epigenetic regulation (Loscher, 2002; Diederich et al., 2010). Beyond epilepsy, VPA has demonstrated therapeutic potential in neurodegenerative disorders, cancer, and psychiatric conditions due to its pleiotropic pharmacological effects.

However, the clinical use of VPA is limited by its well-documented adverse effects, particularly hepatotoxicity and pancreatitis. VPA-induced hepatotoxicity is a severe complication, with an incidence estimated at approximately 1 in 5,000 to 1 in 50,000 patients and is more prevalent in children and individuals with mitochondrial disorders (Jaeschke, 2018; Kadam et al., 2025). Similarly, VPA-associated pancreatitis, is a life-threatening adverse effect and occurs across all age groups. The underlying mechanisms of these toxicities are complex and involve mitochondrial dysfunction, oxidative stress, impaired β-oxidation of fatty acids, accumulation of toxic metabolites, and activation of inflammatory pathways. Dysregulation of key molecular pathways such as oxidative stress signaling, cytokine-mediated inflammation, and apoptotic cascades play a critical role in tissue injury (Bischof et al., 2023).

Despite extensive research on VPA toxicity, the precise molecular targets and interconnected pathways linking hepatotoxicity and pancreatitis remain incompletely understood. Moreover, there is a lack of integrative studies exploring multi-target therapeutic strategies to mitigate these adverse effects. Natural compounds, especially those derived from medicinal plants, have emerged as promising candidates due to their multitarget activity and favorable safety profiles. However, systematic identification of bioactive phytochemicals and their mechanisms against VPA-induced toxicity is still limited. In this context, the present study aims to elucidate the molecular mechanisms underlying VPA-induced hepatotoxicity and pancreatitis using a network pharmacology approach (Li and Zhang, 2013; Shyam and Sabina, 2024). Phytochemicals from *Arctium lappa* were identified through HR-LCMS-QTOF analysis and screened based on ADMET properties. Target prediction, protein–protein interaction analysis, and functional enrichment studies were performed to identify key molecular targets and pathways. Furthermore, molecular docking studies were conducted against key targets, including MAPK8, MCL-1, NLRP3, and TNF-α, to evaluate binding affinity and interaction profiles (Adams and Cooper, 2007; Seki et al., 2012; Swanson et al., 2019). To further validate the stability of these interactions, molecular dynamics simulations were performed, where MCL-1 and NLRP3 complexes demonstrated stable behavior over a 200 ns simulation period. Collectively, this integrative strategy bridges phytochemical profiling with systems-level and molecular insights to provide a comprehensive understanding of the protective potential of *A. lappa* against VPA-induced toxicity. By combining network pharmacology with molecular docking and dynamic simulations, the study not only identifies key targets and pathways involved in hepatotoxicity and pancreatitis but also highlights promising lead compounds with multi-target activity. These findings establish a robust theoretical framework for future experimental validation and may contribute to the development of safer therapeutic interventions for managing VPA-associated adverse effects.

## 2 Materials and methods

*Arctium lappa* (Burdock) powder (Batch No: AV/AL060324) was procured from Kshipra BioTech Pvt. Ltd., India.

### 2.1 Methodology for *Arctium lappa* root extract preparation

To extract naturally occurring chemicals from the selected plant materials, the Soxhlet apparatus was employed to obtain maximum extraction efficiency using organic solvents by hot continuous extraction methods. The *A. lappa* root was dried, ground into a fine powder, and placed into a cellulose extraction thimble that was then positioned in the main chamber of the Soxhlet extractor.

Each of five different organic solvents was tested for its respective solvent-specific efficiency at extracting phytoconstituents utilizing methanol, ethanol, acetone, distilled water, and a hydroalcoholic solvent (50% methanol and 50% water (v/v)). Individual organic solvents were separately placed into round-bottom flasks that are connected to the Soxhlet apparatus-the round-bottom flask heat source will come from a heating mantle. The heating mantle will apply heat to both the bottom of the round-bottom flask and the contents of the round-bottom flask simultaneously causing the organic solvent molecules to vaporize and move from the bottom of the Soxhlet apparatus to the top of Soxhlet apparatus then back to liquid whereupon they will flow down into the cellulose extraction thimble.

Within a few minutes of heating, the solvent in the round-bottom flask began to reflux, enabling efficient extraction of phytochemicals from the plant material. As the solvent vapor condensed and filled the Soxhlet extractor chamber, it reached the overflow level, triggering the siphon mechanism, which transferred the extract back into the round-bottom flask. This continuous cycle of heating, condensation, and siphoning was maintained for 6–8 h until the solvent in the siphon tube became colorless, indicating exhaustive extraction. The collected extracts were then concentrated under reduced pressure using a rotary evaporator to remove the organic solvent. The resulting crude extracts were dried, sealed in amber glass vials, and stored at 4 °C in a refrigerator until further phytochemical and biological analyses. The percentage yield of each extract was calculated using the following formula:

$$\begin{array}{ll} Percentage\;Yield\left(\%\right) & =Weight\;of\;crude\left(g\right)\\ & \;÷Weight\;of\;Plant\;Material\left(g\right)\times 100 \end{array}$$

### 2.2 Phytochemical identification and selection

For metabolite profiling, crude extracts were diluted in methanol and analyzed using an Agilent Velocity Workstation coupled with an Agilent 6550 iFunnel Q-TOF LC/MS system (Agilent Technologies, USA) equipped with a Dual AJS electrospray ionization (ESI) source at the Graduate School of Bioresources, IIT Bombay. Chromatographic separation was achieved on a Hypersil GOLD C18 column (100 × 2.1 mm, 3 μm) using a binary solvent system consisting of 0.1% formic acid in water (Solvent A) and acetonitrile (Solvent B). The column temperature was maintained at 40 °C, and the mobile phase was delivered at a flow rate of 0.3 mL/min over a total run time of 35 min. The gradient program was as follows: 5% B (0–1 min), 100% B (1–25 min), 100% B (25–30 min), and 5% B (31–35 min). A sample volume of 5 μL was injected for each run, and the maximum system pressure was maintained at 1,200 bar.

Mass spectrometry was carried out in positive ESI mode at a drying gas temperature of 250 °C (13 L/min), nebulizer pressure of 35 psig, sheath gas temperature of 300 °C (11 L/min), capillary voltage of 3,500 V, nozzle voltage of 1,000 V, fragmentor voltage of 175 V, skimmer voltage of 65 V, and octopole RF voltage of 750 V. Data was collected in Auto MS/MS mode over a *m/z* range of 120–1,200 at a scan rate of one spectra per second. Precursor ions were chosen based on their relative abundance, with an isolation width of about 4 amu, and active exclusion was enabled to reduce repetitive fragmentation of the same precursor ions. Agilent MassHunter Acquisition Software and Agilent MassHunter Qualitative Analysis Software (Version 12.1) were used to collect and process the data, respectively. Metabolite annotation was accomplished using accurate mass measurements, molecular formula prediction, isotopic pattern evaluation, database-assisted matching, and MS/MS fragmentation analysis. Mass accuracy was measured using the software’s ppm mass error values, and fragment ion validation was carried out by comparing characteristic MS/MS fragmentation patterns to reference databases and published literature. According to the Metabolomics Standards Initiative (MSI), the identified metabolites were classified as Level 2 annotations (putatively annotated compounds) because their identification was supported by spectral matching and fragmentation evidence but not confirmed using authentic reference standards. These potential annotated metabolites were then used for target prediction, network pharmacology, molecular docking, and molecular dynamics studies.

### 2.3 *In-silico* ADME profiling and toxicity assessment

To decrease the likelihood of compounds failing during clinical trials, pharmacokinetic/pharmacodynamic evaluation must be performed on all drugs before late-stage clinical trials that are moving to the next phase. To facilitate this, each of the drugs chosen were collected and placed into a CSV file containing a variety of details, including name, PubChem ID, and SMILES string. Using the Swiss ADME (http://www.swissadme.ch/), the predicted ADME values were generated for each compound (Daina et al., 2017). To screen for inclusion/exclusion of any prospective candidate from the database, we employed stringent screening guidelines: High GI Absorption, Pg Substrate, Lipinski rule of five, and Pain filter. After determining that a compound met all required inclusions/exclusions, it was then evaluated for each of four different types of toxicology using the ProTox 3.0 platform (https://tox.charite.de/protox3/) including Hepatotoxicity, Cytotoxicity, Clinical Toxicity, MMP, Nrf 2, and CYP2C9, CYP2C19, CYP3A3 (Banerjee et al., 2024).

ADMET screening was used to identify phytochemicals with promising projected pharmacokinetic and safety profiles. Pg substrate status was not regarded an independent sign of drug appropriateness; rather, it was assessed as part of the total absorption, distribution, metabolism, and excretion profile. Given the importance of Pg in drug transport, bioavailability, and cellular efflux, both substrate and non-substrate substances may have pharmacological implications depending on the intended therapeutic application. Toxicity filtering was performed using computational predictions of safety-related factors, with substances with lower anticipated toxicity risks being preferred for future investigation. The pathophysiological mechanisms driving VPA-induced hepatotoxicity and pancreatitis were also considered when developing the selection criteria. VPA is extensively metabolized in the liver through glucuronidation, mitochondrial β-oxidation, and CYP-mediated oxidation. Among these routes, CYP-mediated metabolism, particularly through CYP2C9, contributes to the generation of reactive metabolites such 4-ene-VPA and 2,4-diene-VPA, which have been linked to mitochondrial dysfunction, oxidative stress, and hepatocellular injury (Ghodke-Puranik et al., 2013). As a result, CYP interaction patterns were thoroughly assessed during ADMET screening. Compounds expected to have the least inhibitory effects on major CYP isoforms, particularly CYP2C9, CYP2C19, and CYP3A4, were prioritized because they are less likely to interfere with VPA metabolism or raise the likelihood of unfavourable hepatic interactions. These parameters were used together to select phytochemicals with potentially favourable pharmacokinetic properties and enhanced safety profiles for future network pharmacology and molecular modelling studies.

### 2.4 Selection and preparation of ligand library

A total of 111 compounds were analyzed in the Swiss ADME and Protox web servers, and we filtered 22 compounds that were found to follow the selected ADMET parameters. The 3D structure of these compounds was further retrieved from PubChem, a well-known library ligand resource in SDF format. The compounds were further prepared in the Open Babel tool using the MMFF94 force field and saved in PDBQT format (O’Boyle et al., 2011).

### 2.5 Network pharmacology studies

#### 2.5.1 Compound target prediction

Two computational methods that generate ligand-based predictions about the potential molecular targets of the compounds included in the virtual screen were used to predict potential molecular targets for the screened compounds. The first method was Swiss Target Prediction (http://www.swisstargetprediction.ch/), which determined the likely molecular targets based on the molecular structure of the compounds using a structural similarity algorithm (Gfeller et al., 2014). The resultant 15 predicted targets for each compound were then combined and de-duplicated to form List 1. In parallel, the second molecular target prediction tool, Similarity Ensemble Approach (SEA) (https://sea.bkslab.org/). The datasets resulting from this method were then merged, and the duplicate entries were eliminated to establish List 2 (Keiser et al., 2007). Finally, the two lists were compared using a Venn diagram tool (https://bioinformatics.psb.ugent.be/webtools/Venn/) to identify the overlapping genes as the most reliable ligand-based molecular target candidates for subsequent analysis.

#### 2.5.2 Identification of toxicity-associated targets

Genes associated with VPA–induced toxicities were retrieved from two publicly available databases. For hepatotoxicity, gene data were collected from GeneCards (https://www.genecards.org/) and Comparative Toxicogenomics Database (https://ctdbase.org/) (Stelzer et al., 2016; Davis et al., 2025). Duplicate entries were removed from each dataset, and the resulting gene lists were merged to obtain a comprehensive set of hepatotoxicity-related targets. Similarly, genes associated with VPA–induced pancreatitis were obtained from the same databases, followed by the removal of duplicate entries and consolidation into a unified list. Subsequently, a Venn diagram analysis was performed to identify the common targets shared between hepatotoxicity and pancreatitis induced by VPA (List 3).

#### 2.5.3 Identification of common targets

To identify the common genes between phytochemical (ligand-based) predicted targets and VPA–induced toxicity–associated targets obtained from List 3, an intersection analysis was performed. The Venny tool was utilized to determine the shared targets between the compound-predicted datasets and toxicity-associated datasets. The overlapping genes were considered the most relevant ligand–toxicity target candidates and were selected for subsequent analyses.

#### 2.5.4 Protein-protein interaction (PPI) network construction and hub gene identification

The last set of corresponding goals was derived through a Venn diagram comparing anticipated targets based on the ligand-based predicted targets and the target list based on VPA-induced toxicity. The overlapping genes were employed to create a PPI network using the STRING database (https://string-db.org/) (Szklarczyk et al., 2021) and the resulting PPI network was transferred into Cytoscape software (version 3.10.1) (Otasek et al., 2019), for visualization and further network analysis. Subsequently, the CytoHubba plugin in Cytoscape was utilised to determine the most prominent nodes, thereby identifying the top 10 hub targets based on network topology.

#### 2.5.5 Gene ontology and pathway enrichment analysis

Functional annotation of the identified target genes was conducted by means of online tools such as ShinyGO 0.80 (http://bioinformatics.sdstate.edu/go/). An example of this type of analysis includes using ShinyGO to create KEGG pathway maps, biological processes and disease interaction networks for the ten hub target genes (Ge et al., 2020). Finally, a gene ontology (GO) enrichment analysis was performed on the ten hub target genes using the Metascape platform (https://metascape.org/gp/index.html#/main/) and all analyses were completed using a p-value of <0.05 in the *Homo sapiens* species setting to identify significant enriched biological functions and pathways (Zhou et al., 2019).

#### 2.5.6 Selection of protein target

VPA–induced hepatotoxicity and pancreatitis are facilitated by interconnected pathways involving oxidative stress, inflammation, and apoptosis, with critical targets including MAPK8, MCL-1, NLRP3, PTGS1, and TNF-α. When MAPK8 (JNK) is turned on, it causes stress-induced apoptosis in liver and pancreatic cells (El-Mowafy et al., 2016). When the anti-apoptotic protein MCL-1 is turned down, it causes mitochondrial dysfunction and cell death. Simultaneously, the activation of the NLRP3 inflammasome and increased TNF-α levels intensify inflammatory cascades through NF-κB and MAPK signaling pathways, worsening tissue damage. Also, prostaglandin synthesis that happens through PTGS1 (COX-1) makes inflammatory responses even stronger (Bosetti et al., 2003). These targets collectively signify critical molecular mechanisms responsible for VPA-induced hepatic and pancreatic toxicity, justifying their selection for molecular docking and dynamics investigations (Huang et al., 2019; Ezhilarasan and Mani, 2022).

Based on this rationale, five key protein targets were selected, and their crystallographic structures were retrieved from the RCSB Protein Data Bank. The selected proteins and their corresponding PDB IDs were as follows: Mitogen-activated protein kinase 8 (MAPK8; PDB ID: 4QTD), Prostaglandin G/H synthase 1 (PTGS1; PDB ID: 6Y3C), Tumor necrosis factor-α (TNF-α; PDB ID: 9OJO), NACHT, LRR and PYD domains-containing protein 3 (NLRP3; PDB ID: 9GU4), and Myeloid cell leukemia-1 (MCL-1; PDB ID: 5FC4) (Chaikuad et al., 2014; Pelz et al., 2016; Miciaccia et al., 2021; Mackay et al., 2024; Bian et al., 2025). For molecular docking analysis, chain A was selected for MAPK8, MCL-1, NLRP3, and PTGS1, as it represents the active and structurally relevant domain in these proteins. In contrast, TNF-α was considered in its homotrimer form, and all three chains were included in the docking study, as the co-crystallized ligand occupies the central interface of the trimeric complex. Detailed structural and functional characteristics of the selected proteins are provided in Supplementary Table S1.

#### 2.5.7 Molecular docking using AutoDock tools

For the molecular docking analysis, firstly, all the proteins were prepared using the AutoDock tools (Morris et al., 2009). This step consisted of removing any heteroatoms and water molecules while adding polar hydrogen and Kollman charges to each structure. The structures were saved in PDBQT format. For the determination of the active site with each protein, a binding pocket was predicted around the co-crystallized ligands respective to each protein using the ProteinsPlus tool (Schöning-Stierand et al., 2022). Unfortunately for the PTGS1, no co-crystallized ligand was present, so only the predicted binding site was chosen for the PTGS1 target. The details about the predicted residues with each protein, which were used for creating biding site, along with grid size dimensions, have been elaborately mentioned in Supplementary Table S1. With each protein, validation of the docking protocol was also considered by superimposing the co-crystallized with the docked complexes. This step helped to understand whether the ligand is docked in the experimentally determined pocket or not. Finally, the virtual screening of the prepared ligand library, having 22 compounds and the respective co-crystallized, was performed using the AutoDock Vina tool (Trott and Olson, 2010).

#### 2.5.8 Interaction profile analysis

Following the completion of the molecular docking studies, post-docking interactions analyses were performed to assess the docked protein-ligand complexes as per criteria, such as lower docking scores, the type and nature of interactions formed, similarity to interactions seen with reference compounds (co-crystallized ligand), and binding pocket size. PyMOL (https://www.pymol.org/) and Discovery Studio Visualizer (https://www.3ds.com/products/biovia/discovery-studio) were used for visualization and interaction analysis. The most interesting protein-ligand complexes were chosen for further molecular dynamics simulation (MDS) investigations based on these evaluations.

#### 2.5.9 Molecular dynamic simulation (MDS)

Although both proteins and ligands behave dynamically under physiological settings, conventional molecular docking regards the protein as a rigid structure while permitting ligand flexibility. Extended MDS investigations were carried out for the chosen protein-ligand complexes using GROMACS 24.3 software (Abraham et al., 2015) in the ScientiFow prebuilt workflow (https://scientiflow.com/) to overcome this constraint. To evaluate the dynamic behaviour and stability of the complexes in a solvated state, simulations were performed. Topologies for the ligands were generated using the SwissParam online server (Zoete et al., 2011) while those for proteins were done using the CHARMM36 force field and the pdbtogmx utility. Topologies for both the ligands and the proteins were combined manually into a cubic simulation box of 1 nm containing TIP3P water molecules. System neutrality was attained with correct numbers of sodium (Na^+^) and chloride (Cl^−^) ions, followed by solvation and energy minimisation/equilibration of the systems. Energy minimisation was performed over 50,000 steps with restriction of positions for the 100 ps NVT ensembles and 1 bar NPT ensembles at 300 K. Type of production simulation will be run for 200 ns each for all systems with each trajectory recorded for subsequent analyses such as root mean square deviation (RMSD) based upon backbone atoms, root mean square fluctuation (RMSF), radius of gyration (Rg), solvent-accessible surface area (SASA), and number of total hydrogen bonds present in each trajectory.

To explore the motions the proteins (Cα atoms) within the protein–ligand complexes exhibit globally, essential dynamics or Principal Component Analysis (PCA) was employed. PCA reduces the highly complex atomic coordinate data present in a high-dimensional space into a lower-dimensional space; therefore, it allows for the identification of the dominant motions of the system and conformational changes during the simulation and for the interpretation of these complex molecular motions (Ziada et al., 2018). The covariance matrix for the Cα atoms in the ligand-bound state was calculated using the “g_covar” function within GROMACS, enabling the overall mobility of these Cα atoms to be determined. The PCA plots used the first ten eigenvectors because they represent the principal motions of the protein’s motion. The free energy landscapes (FEL) were also analysed using the “g_sham” function to identify energy minima and to evaluate the thermodynamic stability of the various conformational states of the protein throughout the simulation. The interpretation of the results from the MDS studies was carried out based on our previous studies (Sharma and Muniyan, 2024; Jain et al., 2025; Jena et al., 2025).

### 2.6 Binding free energy calculations

The selected protein-ligand complexes were used to calculate the total binding free energy (Molecular Mechanics Poisson-Boltzmann Surface Area (MM-PBSA)) using the gmx_MMPBSA tool (Miller et al., 2012; Valdés-Tresanco et al., 2021). The last 1000 frames of each MDS trajectories were selected for this analysis, and the scores were compared to comment on the stability of different protein-ligand complexes.

## 3 Results and discussion

### 3.1 Extraction yield

The roots of *A. lappa* were extracted using a hydroalcoholic solvent system (methanol: water, 50:50 v/v), yielding an extract with a percentage yield of 12.80% ± 0.15%.

### 3.2 Metabolite profiling of *Arctium lappa* root extract

We used high-resolution liquid chromatography–mass spectrometry coupled with quadrupole time-of-flight (HR-LCMS-QTOF) in both positive and negative ionization modes to analyze the extract. In the negative ionization mode, 88 compounds were found, and in the positive mode, 81 compounds were found. After removing amino acids and duplicate entries, 111 compounds with high signal intensity and identification accuracy over 95% were kept for more study. Supplementary Information File 1 (Supplementary Figure S1) contains the chromatograms corresponding to the data presented in this study.

It should be mentioned that some substances found by HR-LCMS-QTOF analysis, such as ethyl levulinate, carisoprodol, and gabapentin, are not frequently mentioned components of *A. lappa*. Certain identifications may be tentative assignments resulting from spectrum similarity, database overmatching, analytical artifacts, or possible contaminants because compound annotation was mostly dependent on precise mass and database matching. As a result, these annotations should be read cautiously and need to be verified by specific MS/MS tests, reliable reference standards, and further phytochemical research. Thus, the network pharmacology framework serves as a preliminary tool for identifying potential molecular interactions and pathways, providing direction for subsequent *in vitro* and *in vivo* investigations.

### 3.3 *In-silico* pharmacokinetic and toxicity evaluation

Overall, 111 unique metabolites were identified through HR-LCMS-QTOF analysis and subjected to preliminary ADMET screening. Among these, 45 compounds satisfied the selected ADME criteria, including high gastrointestinal (GI) absorption, P-glycoprotein (P-gp) substrate status, compliance with Lipinski’s Rule of Five, and absence of PAINS alerts. Importantly, 22 compounds met all these criteria, supporting their favorable drug-like properties. Further toxicity evaluation using the ProTox 3.0 online platform revealed that these 22 compounds were non-toxic with respect to hepatotoxicity, cytotoxicity, clinical toxicity, mitochondrial membrane potential (MMP) disruption, and nuclear factor erythroid 2–related factor 2 (Nrf2) pathway toxicity. Additionally, these compounds showed no predicted inhibitory effects on key cytochrome P450 enzymes, including CYP2C9, CYP2C19, and CYP3A4. Based on these results, the 22 compounds were selected for further investigation of their potential interactions with target proteins.

### 3.4 Network pharmacology studies

#### 3.4.1 Screening of target genes

**FIGURE 1:**
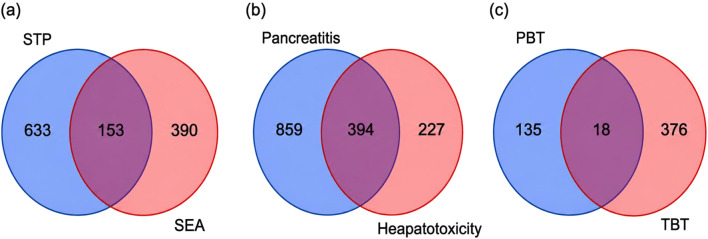
Venn diagram analysis for identification of common targets. **(a)** Overlapping targets predicted by Similarity Ensemble Approach (SEA) and Swiss Target Prediction (STP). **(b)** Common Targets between Hepatotoxicity and Pancreatitis related targets identified from Comparative Toxicogenomic Database (CTD) and GeneCards (GC) databases **(c)** Intersection between Phytochemical-based predicted targets (PBT) and Toxicity-based targets (TBT).

Using Swiss Target Prediction, we predicted the targets for the 22 chosen compounds based on their ligands. We chose the top 15 predicted targets for each molecule. We got 786 unique targets after getting rid of duplicates (List 1). At the same time, the Similarity Ensemble Approach was also used to predict targets, and it found 544 unique targets (List 2). A Venn diagram comparing List 1 and List 2 found 153 common targets (Figure 1a).

To examine targets related to VPA–induced toxicity, genes were obtained from Gene Cards and the Comparative Toxicogenomic Database. After removing duplicates, we put together a list of 620 targets related to VPA induced hepatotoxicity and a list of 1252 targets related to VPA–induced pancreatitis. Using Venny to analyze a Venn diagram, we found 394 common targets between hepatotoxicity and pancreatitis (Figure 1b). Following this, an intersection analysis of phytochemical-derived targets (153) and toxicity-associated targets (394) revealed 18 overlapping targets: CYP1A2, CYP2C19, S1PR1, CASP3, MAPK8, HDAC8, F9, PPARG, AKR1C2, NLRP3, CA3, TNF, CA9, CYP2C9, PTGS1, MCL1, CES1, and HSP90AA1 (Figure 1c). These were deemed key targets for further investigation.

#### 3.4.2 String PPI network

**FIGURE 2:**
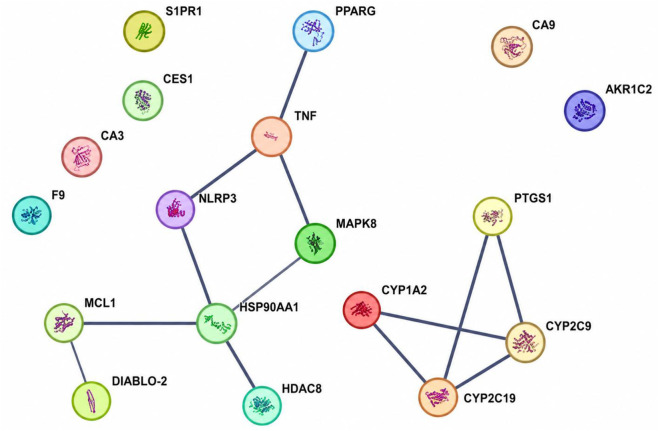
Protein–protein interaction (PPI) network of the common targets constructed using the STRING database. Nodes represent proteins and edges represent their interactions. The network highlights the functional connectivity among the shared targets and provides the basis for identifying key hub proteins involved in valproic acid-induced hepatotoxicity and pancreatitis.

We used the STRING database to make the protein–protein interaction (PPI) network for the 18 common targets. The network that came out of this had 18 nodes and 13 edges. The average node degree was 1.44 and the average local clustering coefficient was 0.352. The PPI enrichment p-value was determined to be 1.46 × 10^−5^, signifying that the observed interactions are significantly greater than what would be anticipated by chance, thus indicating a biologically relevant interaction network (Figure 2).

**FIGURE 3:**
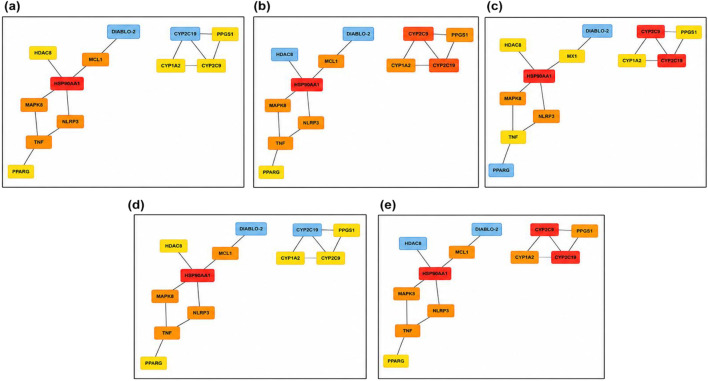
The top ten hub genes were found from the protein-protein interaction (PPI) network using the CytoHubba plugin in Cytoscape. Hub genes were sorted according to network topology using **(a)** bottleneck, **(b)** degree, **(c)** eccentricity, **(d)** closeness, and **(e)** maximal clique centrality (MCC) algorithms. The study identifies critical regulatory genes that may play roles in the molecular pathways driving valproic acid-induced hepatotoxicity and pancreatitis.

The network showed important interactions between targets like TNF, MAPK8, NLRP3, HSP90AA1, and PTGS1. These targets are important for inflammatory and apoptotic pathways that are linked to VPA–induced liver damage and pancreatitis. Then, the built PPI network was brought into Cytoscape (version 3.10.1) for more visualization and topological analysis. We used the CytoHubba plugin and several topological analysis methods, such as maximal clique centrality (MCC), degree, closeness, betweenness (bottleneck), and eccentricity, to find the most important hub genes. These complementary algorithms made sure that key nodes in the network were found reliably. The integrated analysis identified the highest-ranked hub targets, which are expected to be pivotal in mediating VPA–induced hepatotoxicity and pancreatitis. PPI network of the hub targets was shown in Figure 3.

#### 3.4.3 Functional annotation and KEGG pathway analysis

**FIGURE 4:**
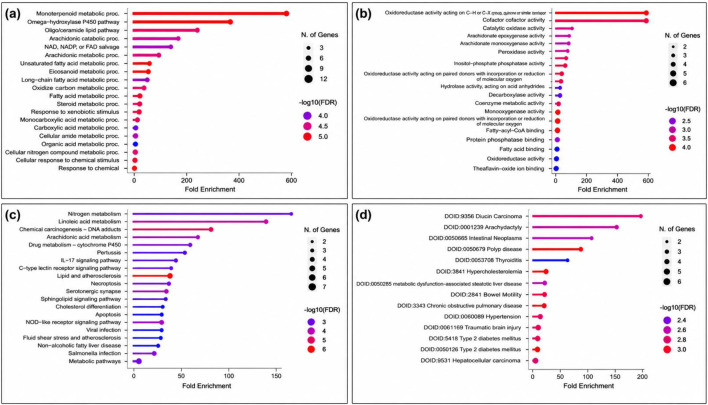
Functional enrichment study of common targets. **(a)** Gene Ontology (GO) Biological Process, **(b)** GO Molecular Function, **(c)** KEGG pathway enrichment, and **(d)** RGD disease enrichment studies. The enriched phrases imply that the targets are involved in metabolic processes, inflammatory responses, and drug metabolism pathways related to valproic acid-induced hepatotoxicity and pancreatitis. The bubble size denotes gene count, color shows statistical significance (−log10 FDR), and x-axis represents fold enrichment. Only terms with an FDR <0.05 were considered significant.

To investigate the biological importance of the chosen target genes, gene ontology (GO) and pathway enrichment studies were carried out (Figure 4). Nitrogen metabolism, linoleic acid metabolism, arachidonic acid metabolism, drug metabolism–cytochrome P450, IL-17 signaling pathway, C-type lectin receptor signaling pathway, necroptosis, apoptosis, NOD-like receptor signaling pathway, sphingolipid signaling pathway, serotonergic synapse, and lipid and atherosclerosis pathways were all significantly involved, according to the KEGG pathway enrichment analysis (Figure 4c). These pathways demonstrate how important inflammation, lipid metabolism, and xenobiotic processing are in the hepatotoxicity and pancreatitis caused by VPA. The results of GO biological process analysis showed that the targets were considerably enriched in processes like the metabolism of monoterpenoids, the omega-hydroxylase P450 pathway, the epoxygenase P450 pathway, xenobiotic catabolic processes, the metabolism of arachidonic acid, the metabolism of fatty acids, the metabolism of steroids, and the response of cells to chemical stimuli (Figure 4a). These results highlight the role of lipid metabolism and metabolic detoxification pathways in drug-induced toxicity. Activities such as oxidoreductase activity (especially acting on CH or CH2 groups), monooxygenase and deacetylase activity, arachidonic acid monooxygenase activity, heme binding, iron ion binding, protein phosphatase binding, and transition metal ion binding were enriched, according to GO molecular function analysis (Figure 4b). The importance of cytochrome P450 enzymes and redox-related pathways in mediating toxicity is further supported by these functions. Significant correlations with ductal carcinoma, intestinal neoplasms, drug allergies, dyslipidemia, steatotic liver disease linked to metabolic dysfunction, acute kidney failure, chemical and drug-induced liver injury, hypertension, type 2 diabetes mellitus, and hepatocellular carcinoma were also revealed by disease enrichment analysis using RGD (Figure 4d). When taken as a whole, our enrichment studies show that the discovered targets are closely associated with inflammatory reactions, metabolic problems, and drug-induced organ toxicity, which supports their significance in hepatotoxicity and pancreatitis caused by VPA.

**FIGURE 5:**
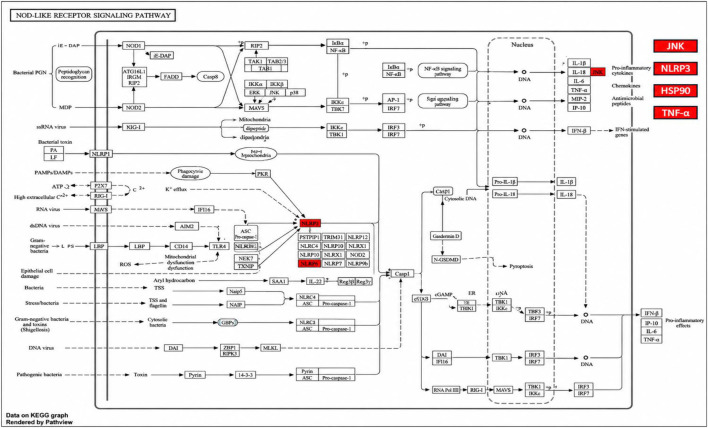
KEGG pathway enrichment analysis of the NOD-like receptor signaling pathway showing the hub targets NLRP3, TNF-α, HSP90AA1, and MAPK8 (JNK). These targets are involved in inflammasome activation, inflammatory signaling, oxidative stress, and apoptosis, suggesting their potential role in the molecular mechanisms of valproic acid-induced hepatotoxicity and pancreatitis. Red-highlighted nodes indicate the identified hub targets.

To clarify the possible molecular mechanisms behind the selected targets, KEGG pathway analysis was carried out; compound-associated genes are indicated in red (Figure 5). The NOD-like receptor signaling pathway was discovered to be especially important among the enriched pathways, underscoring its crucial function in modulating inflammatory and stress responses. Within this circuit, important targets like NLRP3, TNF-α, MAPK8 (JNK), and HSP90AA1 were identified, suggesting their role in cytokine generation, downstream signaling cascades, and inflammasome activation. When the NLRP3 inflammasome is activated, pro-inflammatory cytokines including IL-1β and IL-18 are matured by caspase-1, which contributes to inflammation and cellular damage. Additionally, the engagement of NF-κB and MAPK signaling axis indicates increased transcription of pro-inflammatory mediators, supporting the importance of these pathways in pancreatitis and hepatotoxicity caused by VPA. A coordinated mechanism of toxicity is highlighted by the pathway’s combination of oxidative stress, mitochondrial failure, and immunological activation. Overall, this pathway analysis indicates that the identified substances may modulate inflammasome activation and inflammatory signaling networks to have protective effects. Figure 5 shows a schematic illustration of the suggested mechanism.

#### 3.4.4 Enrichment analysis of all targeted genes

**FIGURE 6:**
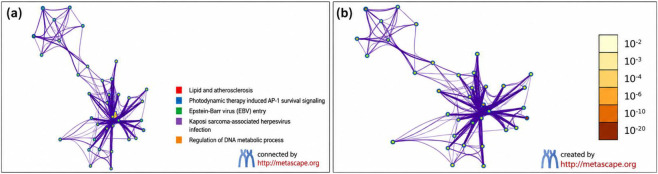
Enrichment network and major MCODE module for VPA-induced toxicity targets. **(a)** Metascape constructed a network of enriched GO words and KEGG pathways (FDR <0.05), with nodes representing functional terms and edges indicating common genes. Nodes are colored based on cluster identity (left) and statistical significance (right). **(b)** The most significant PPI subnetwork found by MCODE (MCODE1), with emphasis on critical modules involved in lipid metabolism, inflammation, and xenobiotic pathways.

**FIGURE 7:**
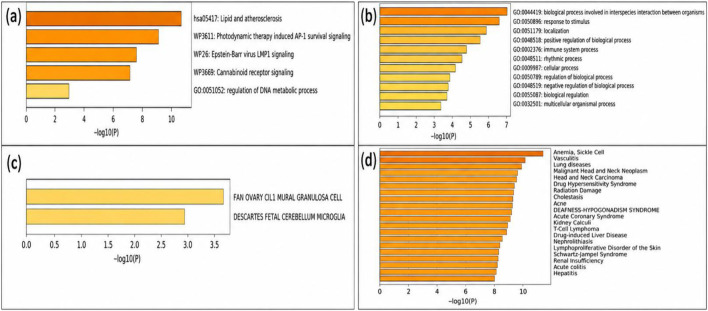
The target gene set is analyzed functionally, transcriptionally, and for disease enrichment. **(a)** The most enriched pathways and functional keywords were found from the KEGG and Gene Ontology (GO) databases. **(b)** GO Biological Process enrichment analysis reveals the most highly enriched biological processes. **(c)** Tissue-specific expression analysis to identify enriched cell types and tissues. **(d)** DisGeNET-based illness enrichment analysis reveals connections with drug-induced liver injury, metabolic disorders, and associated diseases. Colour intensity indicates statistical significance (−log10 P value), with darker hues denoting greater significance.

Significant enrichment of GO keywords, KEGG pathways, illness correlations, and transcriptional regulators associated with the identified target genes was found by Metascape analysis (Figures 6, 7). Immune system functions, sensory response, and biological process regulation were the main biological processes linked to enriched biological processes. Key pathways include lipid and atherosclerosis, hypoxia-inducible factor (HIF-1) signaling, Epstein-Barr virus infection, cannabinoid receptor signaling, and control of DNA metabolic activities were identified using KEGG pathway analysis. With nodes colored according to statistical significance (−log10 P value), the network visualization of enriched phrases showed different functional clusters that reflected closely connected biological processes. Important transcriptional regulators, such as E2F1 and SIRT1, were found through additional investigation, indicating their potential role in controlling the indicated targets. Strong correlations with diseases such drug-induced liver injury, vasculitis, lung diseases, cancer, and metabolic disorders were found using disease enrichment analysis (DisGeNET), indicating the significance of these targets in toxicity-related pathologies. Tissue-specific functional importance was highlighted by gene expression analysis, which revealed enrichment in particular cell types, such as cerebellar microglia and ovarian granulosa cells.

Key functional modules were found using protein–protein interaction network analysis and MCODE clustering; enriched terms included DNA metabolic control, inflammatory signaling, and lipid metabolism. All these results point to the discovered targets’ involvement in interrelated pathways pertaining to inflammation, metabolism, and cellular stress responses, suggesting their possible involvement in pancreatitis and hepatotoxicity caused by VPA.

#### 3.4.5 Selection of protein target

The five selected target’s names and their PDB IDs were Mitogen-activated protein kinase 8 (MAPK8) (PDB ID: 4QTD), Prostaglandin G/H synthase 1 (PTGS1) (PDB ID: 6Y3C), Tumor necrosis factor (TNF- α) (PDB ID: 9OJO), NACHT, LRR and PYD domains-containing protein 3 (NLRP3) (PDB ID: 9GU4), Induced myeloid leukemia cell differentiation protein Mcl-1 (MCL-1) (PDB ID: 5FC4) (Chaikuad et al., 2014; Pelz et al., 2016; Miciaccia et al., 2021; Mackay et al., 2024; Bian et al., 2025). Supplementary Table S1 contains comprehensive information about protein characteristics.

#### 3.4.6 Molecular docking using AutoDock vina

**FIGURE 8:**
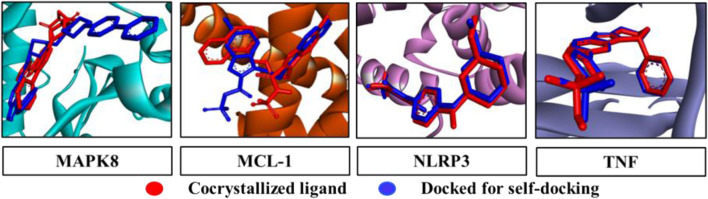
Superimposition of co-crystallized ligand and docked compound for validating the docking protocol across MAPK8, MCL-1, NLRP3, and TNF- α.

The selected targets were prepared using AutoDock tools as mentioned in the methodology section. Before starting the virtual screening, validation of the docking protocol was confirmed by superimposing the co-crystallized ligand with the docked complex. This step helped us to check the docking site of a ligand with the grid size determined in this study. Later, this co-crystallized ligand was used as a reference compound, was used as reference except for the PTGS1 target. For PTGS1, test compounds were compared among themselves. Figure 8 shows the visual representation of the docking protocol for the protein targets MAPK8, MCL-1, NLRP3, and TNF- α. We could see the proximity of superimposed structures, and the RMSD values of all these cases were nearby 2Å, which validated our docking protocol for this study. After validating the docking protocol, a total of 22 compounds, along with the respective crystallized ligands, were docked with all five targets. The docked complexes were analyzed for post-docking analysis in the next step.

#### 3.4.7 Interaction profile analysis

**FIGURE 9:**
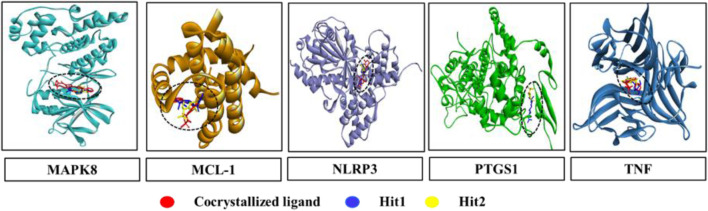
Superimposition of Hit1, Hit2, and Co-crystallized ligand (reference) to confirm the similar binding site across MAPK8, MCL-1, NLRP3, PTGS1, and TNF- α.

All the protein-ligand complexes were analyzed using DSV and Pymol. Supplementary Table S2 describes the complete docking score of the ligand library, and overall docking scores ranged from −12.8 kcal/mol to −3.5 kcal/mol across the selected five proteins. Upon comparing all the test compounds with the co-crystallized ligand as a reference, it was identified that none of the test compounds exhibited a docking score lower than the reference on any of the targets. That’s why the scores of the test compounds were compared among themselves, and the top two ligands were identified, which performed well on multiple targets. Overall, the top two compounds selected were, i.e., 4-Hydroxy-3-methoxy-2,10-bisaboladien-9-one (Hit1) and Fabianine (Hit2), which outperformed on multiple targets. Hit1 outperformed on MAPK8, MCL-1, NLRP3, and TNF- α, whereas Hit2 performed well on MAPK8, PTGS1 and TNF- α. The superiority of Hit1 and Hit2 was decided based on multiple target performance in the molecular docking stage and the lowest docking score among the other test compounds. Figure 9 shows the superimposition of Hit1, Hit2, and the co-crystallized ligand (reference) to confirm the similar binding site across MAPK8, MCL-1, NLRP3, PTGS1, and TNF- α. This analysis also confirmed that Hit1 and Hit2 are occupying the same binding site as that of the co-crystallized ligand, except for PTGS1. Both Hit1 and Hit2 were further analyzed through an interaction profile to confirm the type and number of H-bonds occupied by them in the binding pocket of the selected targets.

**FIGURE 10:**
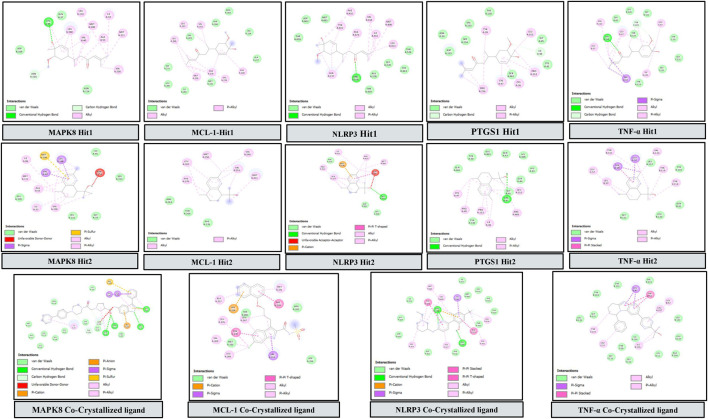
2D interaction profile of Hit1, Hit2 and Co-crystallized ligand with MAPK8, MCL-1, NLRP3, PTGS1, and TNF- α.

Table 1 and Figure 10 show the interaction profile of Hit1, Hit2, and the co-crystallized ligand bound with MAPK8, MCL-1, NLRP3, PTGS1, and TNF- α. For instance, MAPK8-Co-crystallized ligand complex attained a docking score of −12.1 kcal/mol upon establishing conventional H-bonds (H-bonds) with residues ALA36, GLN37, LYS55, GLU73, and carbon-hydrogen bonds (C-H bonds) with residues SER155 and ASN156. Additionally, Van der Waals (VD) interactions were observed with residues GLY33, SER34, GLY35, ARG69, LEU110, MET11, APS112, ALA113, ASN114, LYS153, VAL158, and Pi-interactions with residues ILE32, ALA36, VAL40, LYS55, MET108, LEU168, and ASP169. Like the co-crystallized ligand, Hit1 formed an identical H-bond with residues LYS55 and a C-H bond with residue ASN156, with a docking score of −7.6 kcal/mol in the binding pocket of the MAPK8 protein. Additionally, VD interacts with residues GLN37, ASP114, ASP169 and Pi-interactions with residues ILE32, VAL40, ALA53, MET108, LEU110, MET111, VAL158, LEU168 were also seen in the same MAPK8-Hit1 complex. In contrast, complex MAPK8-Hit2, a docking score of −7.6 kcal/mol was seen without formation of any h-bonds. However, some VD interactions with residues GLY33, LYS55, GLU109, LEU110, SER155, and Pi-interactions with residues ILE32, VAL40, ALA53, ILE86, MET108, MET111, VAL158, and LEU168 were also seen, which confirmed the position of the same binding location as that of the co-crystallized ligand. Order performance based on docking analysis could be co-crystallized ligand being superior, followed by Hit1 and Hit2 with the MAPK8 protein.

**TABLE 1:** Docking score (kcal/mol) of the test library with selected targets.

Complex	Score (kcal/mol)	Conventional H-bonds	C-H bond	VD	Pi-interactions	Others
MAPK8-Hit1	−7.6	LYS55	ASN156	GLN37, ASP114, ASP169	ILE32, VAL40, ALA53, MET108, LEU110, MET111, VAL158, LEU168	—
MAPK8-Hit2	−7.6	—	—	GLY33, LYS55, GLU109, LEU110, SER155	ILE32, VAL40, ALA53, ILE86, MET108, MET111, VAL158 LEU168	ASN114
MAPK8-Co-crystallized ligand	−12.1	ALA36, GLN37, LYS55, GLU73	SER155, ASN156	GLY33, SER34, GLY35, ARG69, LEU110, MET11, APS112, ALA113, ASN114, LYS153, VAL158	ILE32, ALA36, VAL40, LYS55, MET108, LEU168, ASP169	ASN156
MCL-1-Hit1	−6.6	—	—	HIS224, ALA227, MET231, VAL249, ARG263, THR266, GLY271, LEU290, ILE294	PHE228, LEU246, MET250, VAL253, LEU267, PHE270, VAL274	
MCL-1-Hit2	−5.5	—	—	PHE228, ARG263, THR266	MET231, VAL249, MET250, VAL253, LEU267, PHE270	
MCL-1-Co-crystallized ligand	−8.3	—	—	MET250, ASP256, ARG263, THR266	HIS224, ALA227, PHE228, MET231, LEU235, LEU246, VAL249, VAL253, LEU267, PHE270	
NLRP3-Hit1	−7.5	ARG578	—	ALA228, GLY229, THR439, TYR443, THR524, THR659, MET661, ASP662	ALA227, MET408, PHE410, ILE411, LEU413, VAL414, PHE575, TYR632	—
NLRP3-Hit2	−6.2	THR659	—	PHE575, SER658, ASP662	ALA227, PRO352, PHE410, ILE411, ARG578, TYR632, MET661	TYR632
NLRP3-Co-crystallized ligand	−10.7	ALA228, ARG578	—	MET408, PHE410, LEU413, ILE417, THR439, THR524, ILE574, PHE579, SER626, GLU629, ASP662	ALA272, VAL353, ILE411, VAL414, TYR443, PHE575, ARG578, LEU628, TYR632, MET661	
PTGS1-Hit1	−7.0	—	ILE46	ASN34 CYS41, GLY45, TYR130, ASP135, SER154, VAL155, GLN461	CYS36, TYR39, CYS47, LEU152, PRO153, PRO156	—
PTGS1-Hit2	−7.9	CYS41	—	TYR39, GLN42, HIS43, GLN44, GLY45, TYR130, GLN461, GLU465, LYS468	PRO40, CYS41, ILE46, CYS47, LEU152, PRO153, ARG469	
PTGS1-Co-crystallized ligand	NA	NA	NA	NA	NA	NA
TNF- α -Hit1	−8.7	TYR: B151	TYR: C119	TYR: A119, SER: B60, TYR: B119, LEU: B120, GLY: B121, TYR: C59, GLY: C121, GLY: C122	LEU: A57, VAL: A123, ILE: A155, LEU: B57, TYR: B59, TYR: B151, LEU: C57, TYR: C119	—
TNF- α -Hit2	−8.6	—	—	TYR: A119, LEU: A157, LEU: B120, GLN: B61, GLY: B121	LEU: A57, VAL: A123, LEU: B57, TYR: B59, TYR: B119, TYR: B151, LEU: C57, TYR: C119	—
TNF- α -Co-crystallized ligand	−12.8	—	—	LYS: A11, VAL: A123, ILE: A155, ALA: A156, TYR: B119, LEU: B120, GLY: B121 TYR: B151, LEU: B157, TYR: C59, TYR: C121, GLY: C122 LEU: C157	LEU: A57, LEU: A157 LEU: B57 TYR: B59, ILE: B155, LEU: C57, TYR: C119	

On the MCL-1 target, the MCL-1-Co-crystallized ligand complex exhibited a docking score of −8.3 kcal/mol due to hydrophobic interactions such as VD interactions with residues MET250, ASP256, ARG263, THR266, and Pi-interactions with residues HIS224, ALA227, PHE228, MET231, LEU235, LEU246, VAL249, VAL253, LEU267, and PHE270. Similarly, the MCL-1-Hit1 complex attained a docking score of −6.6 kcal/mol upon forming VD interactions with residues HIS224, ALA227, MET231, VAL249, ARG263, THR266, GLY271, LEU290, ILE294, and Pi-interactions with residues PHE228, LEU246, MET250, VAL253, LEU267, PHE270, and VAL274. Lastly, MCL-1-Hit2 complex achieved a docking score of −5.5 kcal/mol by forming VD interactions with residues PHE228, ARG263, THR266, and Pi-interactions with residues MET231, VAL249, MET250, VAL253, LEU267, and PHE270. Notably, none of the complexes formed an H-bond upon binding with the MCL-1 target. These complexes might be stabilized because of hydrophobic interactions alone. The order of stabilization based on docking score could be predicted as Co-crystallized ligand being better followed by Hit1 and Hit2.

On the target NLRP3, the NLRP3-Co-crystallized ligand complex had a docking score of −10.7 kcal/mol upon forming H-bonds with residues ALA228 and ARG578. The other interactions, VD interactions with residues MET408, PHE410, LEU413, ILE417, THR439, THR524, ILE574, PHE579, SER626, GLU629, ASP662 and Pi-interactions with residues ALA272, VAL353, ILE411, VAL414, TYR443, PHE575, ARG578, LEU628, TYR632, and MET661 were also seen in the same complex. Comparatively, the NLRP3-Hit1 complex attained a docking score of −7.5 kcal/mol by forming the same H-bond as that of the co-crystallized ligand with residues ARG578. This marked the significance of Hit1 on the NLRP3 target. Other VD interactions with residues ALA228, GLY229, THR439, TYR443, THR524, THR659, MET661, ASP662 and Pi-interactions with residues ALA227, MET408, PHE410, ILE411, LEU413, VAL414, PHE575, and TYR632 were also observed simultaneously. Lastly, NLRP3-Hit2 complex achieved slightly higher docking score (−6.2 kcal/mol) than other two complexes by forming an H-bond with residues THR659 along with VD interactions with residues PHE575, SER658, ASP662, and Pi-interactions with residues ALA227, PRO352, PHE410, ILE411, ARG578, TYR632, and MET661. Based on docking analysis, we could predict the stability as the Co-crystallized ligand being most stable, followed by Hit1 and Hit2 in the binding pocket of the NLRP3 protein.

There was no co-crystallized ligand on the PTGS1 protein. Therefore, we compared the test compounds among themselves for the PTGS1 protein. For example, the PTGS1-Hit1 complex attained a docking score of −7.0 kcal/mol, a C-H bond with residues ILE46, along with VD interactions with residues ASN34, CYS41, GLY45, TYR130, ASP135, SER154, VAL155, GLN461, and Pi-interactions with residues CYS36, TYR39, CYS47, LEU152, PRO153, and PRO156. On the flip side of the coin, PTGS1-Hit2 complex achieved a docking score of −7.9 kcal/mol by forming H-bonds with residues CYS41 along with VD interactions with residues TYR39, GLN42, HIS43, GLN44, GLY45, TYR130, GLN461, GLU465, LYS468, and Pi-interactions with residues PRO40, CYS41, ILE46, CYS47, LEU152, PRO153, and ARG469. Notably, Hit2 performed superior to all other test compounds which the order of stability as Hit2 being more stable than Hit1 based on docking studies.

Lastly, on target TNF α, we considered three homotrimer chains A, B, and C, since it was found to be active only in the trimer state. Also, the co-crystallized ligand was located at the center of all three chains. The docking score of the co-crystallized ligand in the binding pocket of TNF- α protein was found to be −12.8 kcal/mol due to VD interactions with residues LYS: A11, VAL: A123, ILE: A155, ALA: A156, TYR: B119, LEU: B120, GLY: B121, TYR: B151, LEU: B157, TYR: C59, TYR: C121, GLY: C122, LEU: C157 and Pi-interactions with residues LEU: A57, LEU: A157, LEU: B57, TYR: B59, ILE: B155, LEU: C57, and TYR: C119. Apparently, TNF- α -Hit1 complex attained a docking score of −8.7 kcal/mol by forming an H-bond with residues TYR: B151 and a C-H bond with residue TYR: C119. Additionally, VD interactions with residues TYR: A119, SER: B60, TYR: B119, LEU: B120, GLY: B121, TYR: C59, GLY: C121, GLY: C122 and Pi-interactions with residues LEU: A57, VAL: A123, ILE: A155, LEU: B57, TYR: B59, TYR: B151, LEU: C57, TYR: C119 were also found in the same complex. Lastly, a docking score of −8.6 kcal/mol in complex TNF- α -Hit2 was also seen due to VD interactions with residues TYR: A119, LEU: A157, LEU: B120, GLN: B61, GLY: B121, and Pi-interactions with residues LEU: A57, VAL: A123, LEU: B57, TYR: B59, TYR: B119, TYR: B151, LEU: C57, and TYR: C119. Based on the docking score, it could be said that co-crystallized performed well, followed by Hit1 and Hit2.

Combining the docking studies of test compounds specifically, Hit1 and Hit2, we can conclude that the co-crystallized ligand outperformed on target MAPK8, MCL-1, NLRP3, and TNF- α. Furthermore, among the two selected hit compounds for interaction profile analysis, Hit1 exhibited better stability on protein targets, MAPK8, MCL-1, NLRP3, and TNF- α than Hit2. On the other hand, Hit2 outperformed the PTGS1 target dominantly. To achieve multi-target, Hit1 was further selected for further investigation than Hit2. Therefore, based on the predictions done with docking studies, the co-crystallized ligand and Hit1 bound with MAPK8, MCL-1, NLRP3, and TNF- α were shortlisted for the MDS studies.

### 3.5 Molecular dynamic simulation (MDS)

To understand the nature of complexes in dynamic environments, a 200 ns MDS study for the complexes MAPK8-Hit1, MAPK8-Co-crystallized ligand, MCL-1-Hit1, MCL-1 Co-crystallized ligand, NLRP3-Hit1, NLRP3-Co-crystallized ligand, TNF- α -Hit1, and TNF- α -Co-crystallized ligand was performed using GROMACS 24.3 version. Each trajectory runs were thoroughly analyzed using RMSD, RMSF, RG, SASA, number of H-bonds, etc.

#### 3.5.1 RMSD trajectory analysis

**FIGURE 11:**
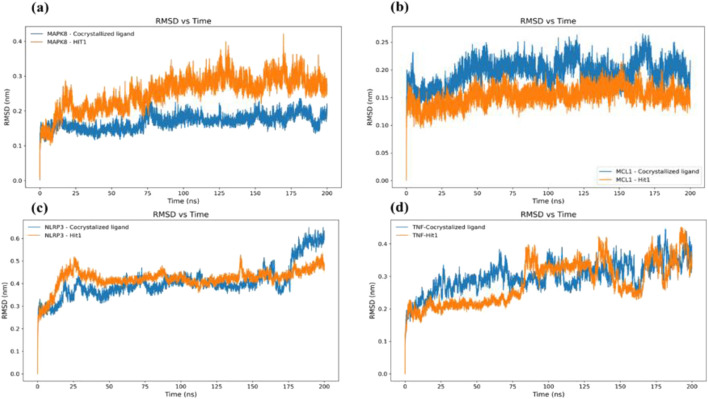
Representation of the RMSD trajectory of the Co-crystallized ligand and Hit1 in the binding pocket of **(a)** MAPK8, **(b)** MCL-1, **(c)** NLRP3, and **(d)** TNF- α proteins.

RMSD analysis defines the changes in the position of the backbone atom from the initial state. Lower RMSD values correspond to the stable protein-ligand system. On the MAPK8 target, the RMSD trajectories ranged from 0.1 nm to 0.45 nm MAPK8-Hit1complex exhibited a higher RMSD plot with a major fluctuation at 20 ns, along with some other minor fluctuations, whereas MAPK8-Co-crystallized ligand complex attained a completely stable RMSD plot. The calculated average RMSD values were found to be higher for MAPK8-Hit1 (0.2527 ± 0.0480 nm) than for MAPK8-Co-crystallized ligand (0.1685 ± 0.0201 nm). This confirmed the superior nature of the co-crystallized ligand over Hit1 in the binding pocket of the MAPK8 protein (Figure 11a).

In contrast, on the MCL-1 target, the ranges of the RMSD plots were found between 0.10 nm and 0.25 nm. Here, the RMSD plots were superimposed on each other, but MCL-1-Hit1 attained a lower RMSD trajectory than the MCL-1-Co-crystallized ligand. The calculated average RMSD values were found to be 0.1526 ± 0.0160 nm and 0.1947 ± 0.0227 nm for MCL-1-Hit1 and MCL-1-Co-crystallized ligand, respectively. Based on the RMSD analysis, we could predict the better stability of Hit1 than the co-crystallized ligand in the binding pocket of MCL-1 protein (Figure 11b).

On the NLRP3 target, the RMSD plots ranged from 0.23 nm to 0.65 nm. The RMSD plots for both complexes increase till 25 ns (equilibration time), after which both plots become constant till 170 ns. At 170 ns, the NLRP3-Co-crystallized ligand complex had a major peak till 200 ns. The average RMSD values were estimated as 0.4219 ± 0.0417 nm and 0.4058 ± 0.0728 nm for NLRP3-Hit1 and NLRP3-Co-crystallized ligand. Based on the RMSD profile, a similar behavior of both Hit1 and the co-crystallized ligand could be marked in the binding pocket of the NLRP3 protein (Figure 11c).

Lastly, on the TNF- α target, 0.17 nm to 0.45 was the range of RMSD plots. Notably, both complexes remained stable till 80 ns; after that, several major and minor fluctuations were observed in both trajectories. The estimated average RMSD values for TNF- α _Hit1 and TNF- α -Co-crystallized ligand complexes were found as 0.2801 ± 0.0660 nm and 0.3001 ± 0.0463 nm, respectively. Based on the RMSD analysis, we could say that the Hit1 compound achieved comparative stability in the binding pocket of the TNF- α protein compared to the co-crystallized ligand (Figure 11d).

#### 3.5.2 RMSF trajectory analysis

**FIGURE 12:**
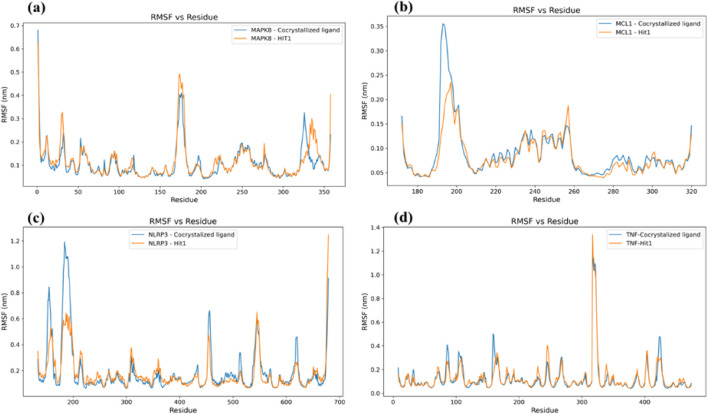
Representation of the RMSF trajectory of the Co-crystallized ligand and Hit1 in the binding pocket of **(a)** MAPK8, **(b)** MCL-1, **(c)** NLRP3, and **(d)** TNF- α proteins.

The RMSF analysis provides the residual-level fluctuations in a protein upon binding with ligand molecules. In general, lower RMSF values indicate a stable protein-ligand complex. On the MAPK8 target, the RMSF plots ranged from 0.04 nm to 0.5 nm. The calculated average RMSF values were 0.1145 ± 0.0788 nm and 0.1065 ± 0.0698 nm for MAPK8-Hit1 and MAPK8-Co-crystallized ligand, respectively. The overlapping RMSF plots for both the complexes and the closer average RMSF values justify the similar nature of Hit1 and the co-crystallized ligand in the binding pocket of the MAPK8 protein (Figure 12a).

On the MCL-1 target, 0.025 nm–0.37 nm was the range of the RMSF plots. Notably, residues in the range of 191–195 achieved the highest RMSF values till 0.37, whereas the same region of much stabilization upon binding with the Hit1 molecules. However, average RMSF values of MCL-1-Hit1 (0.0824 ± 0.0397 nm) and MCL-1-Co-crystallized ligand (0.0897 ± 0.0559 nm) defined the close behavior of the Hit1 and co-crystallized ligand in the binding pocket of MCL-1 protein (Figure 12b).

On the NLRP3 target, the RMSF plots ranged from 0.05 nm to 1.2 nm. Residues between 150 and 200 were highly fluctuating, which also stabilized upon binding with Hit1 more than the co-crystallized bound state. The estimated average RMSF values were identified as 0.1755 ± 0.1350 nm and 0.1879 ± 0.1868 nm for NLRP3-Hit1 and NLRP3-Co-crystallized ligand, respectively. Overall, we could see a slightly superior nature of Hit1 over the co-crystallized ligand in the binding pocket of the NLRP3 protein (Figure 12c).

On the TNF- α target, from 0.05 nm to 1.35 nm was the ranges of RMSF plots. The calculated average RMSF values were found to be 0.1280 ± 0.1362 nm and 0.1247 ± 0.1399 nm for TNF- α -Hit1 and TNF- α -Co-crystallized ligand, respectively. Again, a similar nature of Hit1 and the co-crystallized ligand could be seen with the TNF- α protein (Figure 12d).

#### 3.5.3 RG trajectory analysis

**FIGURE 13:**
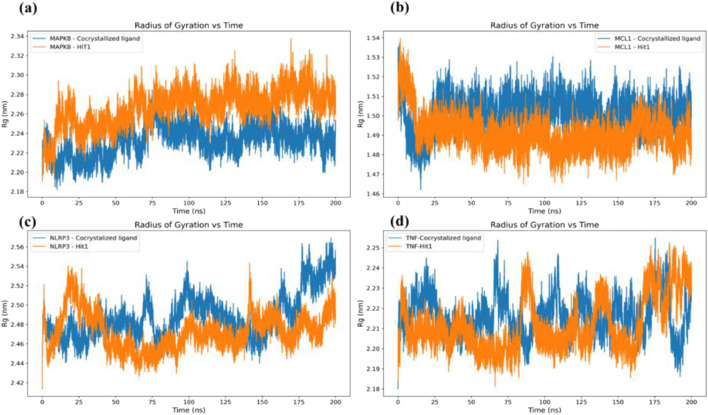
Representation of the RG trajectory of the Co-crystallized ligand and Hit1 in the binding pocket of **(a)** MAPK8, **(b)** MCL-1, **(c)** NLRP3, and **(d)** TNF- α proteins.

The RG parameter defines the compactness of a protein-ligand system. Lower RG values will define a more compact and more stable protein-ligand system. On the MAPK8 target, RG plots showed similar trajectory trends for Hit1 and the co-crystallized ligand, and average RG values of 2.2667 ± 0.0193 nm and 2.2326 ± 0.0155 nm for MAPK8-Hit1 and MAPK8-Co-crystallized ligand, respectively. Though the average RG values of both the complexes are closer, the plot in Figure 13a shows the lower trajectory trends for the co-crystallized ligand than the Hit1, which marks the superior nature of the co-crystallized ligand than the Hit1 in the binding pocket of MAPK8.

On the MCL-1 target, the estimated average RG values for MCL-1-Hit1 and MCL-1-Co-crystallized ligand were found to be 1.4912 ± 0.0091 nm and 1.5015 ± 0.0083 nm, respectively. The average RG values and the RG plots trends confirmed the stability of Hit1 compared to the co-crystallized ligand in the binding pocket of MCL-1 protein (Figure 13b).

On the NLRP3 target, the calculated average RG values for NLRP3-Hit1 and NLRP3-Co-crystallized ligand complexes were found to be 2.4728 ± 0.0188 nm and 2.4877 ± 0.0229 nm, respectively. The average RG values of these complexes were close to each other, but the patterns in the RG plots, as shown in Figure 13c, confirmed the slight superiority of Hit1 over the co-crystallized ligand in the binding pocket of NLRP3 protein.

On the TNF- α protein, like the RMSD plots, RG plots also exhibited several major and minor fluctuations. The calculated average RG values were found to be 2.2117 ± 0.0130 nm and 2.2162 ± 0.0096 nm for TNF- α _Hit1 and TNF- α -Co-crystallized ligand complexes, respectively. The closer average RG values and fluctuating RG plots prevented us from commenting on the stability of either of the complexes (Figure 13d).

#### 3.5.4 SASA trajectory analysis

**FIGURE 14:**
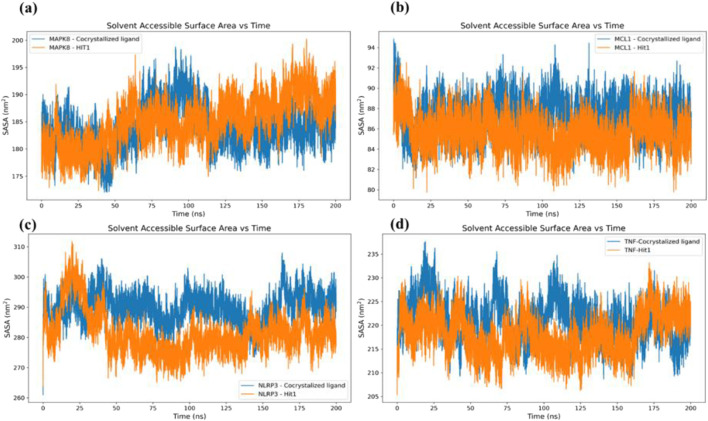
Representation of the SASA trajectory of the Co-crystallized ligand and Hit1 in the binding pocket of **(a)** MAPK8, **(b)** MCL-1, **(c)** NLRP3, and **(d)** TNF- α proteins.

The SASA analysis determines the accessibility of the portion of the macromolecules, such as protein to the water. A lower SASA value corresponds to a stable protein-ligand system. On the MAPK8 target, the estimated SASA values of 185.0263 ± 4.2772 nm^2^ and 183.8761 ± 3.8444 nm^2^ for MAPK8-Hit1 and MAPK8-Co-crystallized ligand complexes clearly defined the greater stability of the co-crystallized ligand than Hit1 in the binding pocket of the MAPK8 target (Figure 14a).

On the MCL-1 target, 85.6729 ± 1.6907 nm^2^ and 87.2391 ± 1.6895 nm^2^ were the calculated average SASA values for MCL-1-Hit1 and MCL-1-Co-crystallized ligand complexes, respectively. This clearly defined the greater stability of the Hit1 than the co-crystallized ligand in the binding pocket of MCL1 protein (Figure 14b).

Similarly, on the NLRP3 target, the average SASA values differences for NLRP3-Hit1 (281.3944 ± 6.9113 nm^2^) and NLRP3-Co-crystallized ligand (289.8352 ± 4.7982 nm^2^) clearly justified the greater stability of the Hit1 than the co-crystallized ligand upon binding with the NLRP3 target (Figure 14c). Lastly, on the TNF- α target, the calculated SASA values were found to be 218.1339 ± 4.1217 nm^2^ and 221.1040 ± 4.1415 nm^2^ for TNF- α -Hit1 and TNF- α -Co-crystallized ligand, respectively. Based on the SASA analysis, we could define the greater stability of Hit1 than the c0-crystallized ligand in the binding pocket of the TNF- α protein (Figure 14d).

#### 3.5.5 H-bonds trajectory analysis

**FIGURE 15:**
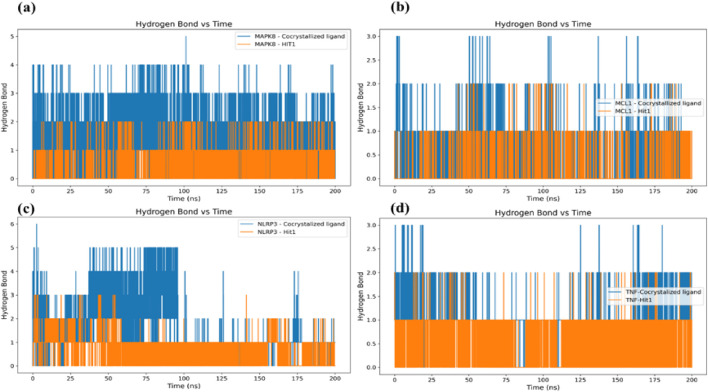
Representation of the H-bonds trajectory of the Co-crystallized ligand and Hit1 in the binding pocket of **(a)** MAPK8, **(b)** MCL-1, **(c)** NLRP3, and **(d)** TNF- α proteins.

More H-bonds in protein-ligand system complexes define their greater stability. The calculated number of H-bonds has been shown in Figures 15a–d, where MAPK8-Hit1 (0–2), MAPK8-Co-crystallized ligand (0–5), MCL-1-Hit1 (0–2), MCL-1-Co-crystallized ligand (0–3), NLRP3-Hit1 (0–3), NLRP3-Co-crystallized ligand (0–6), TNF- α _Hit1 (0–2), and TNF- α -Co-crystallized ligand (0–3). Notably, Hit1 did not acquire more H-bonds than the co-crystallized ligand on any of the targets, but the other analysis shows the high stability of Hit1 on MCL-1 and NLRP3 targets, and moderate stability on the TNF- α target. This could be due to hydrophobic interactions responsible for the complex’s stability. However, persistent and moderate H-bonds could be seen in all the Hit1-bound complexes. Table 2 shows the average RMSD, RMSF, RG, SASA, and number of H-bonds analyzed in this study.

**TABLE 2:** Average values of RMSD, RMSF, RG, SASA, and number of H-bonds.

Protein-ligand complexes	RMSD (nm)	RMSF (nm)	RG (nm)	SASA (nm^2^)	H-bonds	Total binding energy (kcal/mol)
MAPK8-Hit1	0.2527 ± 0.0480	0.1145 ± 0.0788	2.2667 ± 0.0193	185.0263 ± 4.2772	0–2	−26.33 ± 2.43
MAPK8-Co-crystallized ligand	0.1685 ± 0.0201	0.1065 ± 0.0698	2.2326 ± 0.0155	183.8761 ± 3.8444	0–5	−38.87 ± 4.21
MCL-1-Hit1	0.1526 ± 0.0160	0.0824 ± 0.0397	1.4912 ± 0.0091	85.6729 ± 1.6907	0–2	−23.92 ± 2.90
MCL-1-Co-crystallized ligand	0.1947 ± 0.0227	0.0897 ± 0.0559	1.5015 ± 0.0083	87.2391 ± 1.6895	0–3	−23.49 ± 3.05
NLRP3-Hit1	0.4219 ± 0.0417	0.1755 ± 0.1350	2.4728 ± 0.0188	281.3944 ± 6.9113	0–3	−21.14 ± 2.67
NLRP3-Co-crystallized ligand	0.4058 ± 0.0728	0.1879 ± 0.1868	2.4877 ± 0.0229	289.8352 ± 4.7982	0–6	−21.72 ± 3.25
TNF_Hit1	0.2801 ± 0.0660	0.1280 ± 0.1362	2.2117 ± 0.0130	218.1339 ± 4.1217	0–2	−35.52 ± 2.20
TNF-Co-crystallized ligand	0.3001 ± 0.0463	0.1247 ± 0.1399	2.2162 ± 0.0096	221.1040 ± 4.1415	0–3	−36.15 ± 2.62

#### 3.5.6 PCA and FEL analysis

For further investigation, additional PCA and FEL analysis were done for MAPK8-Hit1, MAPK8-Co-crystallized ligand, MCL-1-Hit1, MCL-1-Co-crystallized ligand, NLRP3-Hit1, NLRP3-Co-crystallized ligand, TNF- α -Hit1, and TNF- α -Co-crystallized ligand complexes. The methodology section provides a detailed description of the technique and goal of this investigation. An unstable protein-ligand complex PCA plot typically has several minima energy basins, whereas a stable protein-ligand complex PCA plot typically has a single blue peak (energy minima basin). This is further explained by the fact that several minimum energy basins are constantly searching for different protein conformations. The investigation will compare the Hit1 compound with the co-crystallized ligand based on the number of energy basins (blue peaks) that develop in a protein-ligand interaction. For instance, on the MAPK8 target, in the MAPK8-Hit1 complex, one deep blue region is seen, whereas in the MAPK8-Co-crystallized ligand complex, two deep blue peaks were seen, which marked the superiority of Hit1 over the co-crystallized ligand in the pocket of the MAPK8 target protein. However, this trend was opposite to that seen in the previous analysis for the MAPK8 target.

**FIGURE 16:**
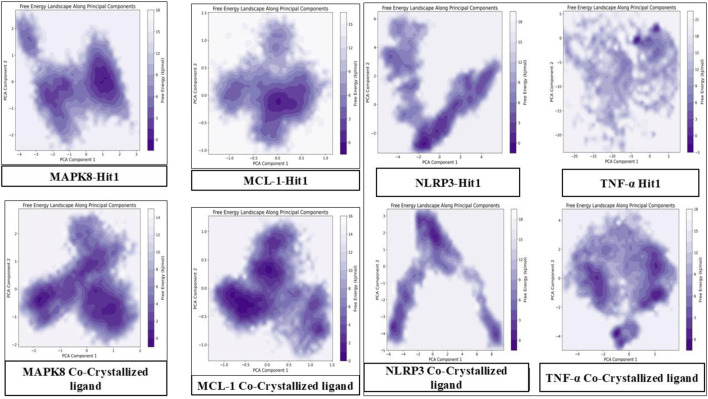
2D FEL plots along with PCA of Hit1 and the co-crystallized ligand in the binding pocket of MAPK8, MCL-1, NLRP3, and TNF- α.

**FIGURE 17:**
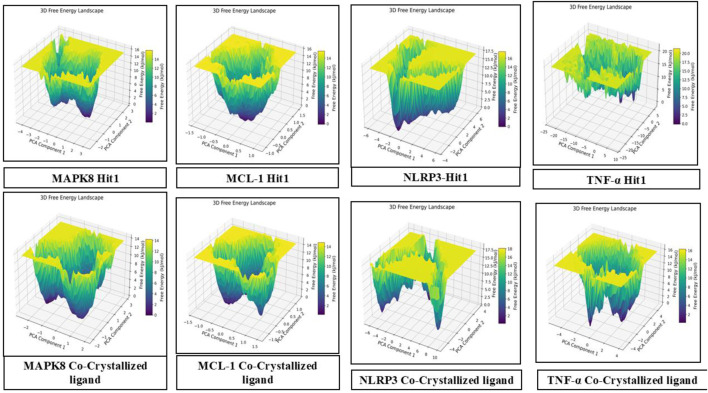
3D Fel plot analysis of Hit1 and the co-crystallized ligand in the binding pocket of MAPK8, MCL-1, NLRP3, and TNF- α.

On the MCL-1 target, like previous analysis, the MCL-1-Hit1 complex attained a single deep blue peak, whereas the MCL-1-Co-crystallized ligand complex had three deep blue regions. This marked the significance of Hit1 over the co-crystallized ligand in the binding pocket of the MCL-1 protein. On the NLRP3 target, diverse conformational plots were seen for both complexes. However, one blue peak was observed in the NLRP3-Hit1 complex, whereas two blue peaks were observed in the NLRP3-Co-crystallized ligand complex. This confirmed the similar nature due to the similar conformational space of Hit1 and the co-crystallized ligand in the binding pocket of the NLRP3 protein. Lastly, on the TNF- α target, neither Hit1 nor the co-crystallized ligand showed a distinct deep blue peak region. Both complexes, TNF- α -Hit1 and TNF- α -Co-crystallized ligand, attained multiple conformations throughout the simulation period (Figure 16). A similar trend was observed in 3D FEL plots also, as shown in Figure 17. Overall, based on the 2D FEL along with PCA and 3D FEL investigations, we could find that Hit1 outperformed the co-crystallized ligand with higher stability with MCL-1 and NLRP3 proteins and least stability with TNF- α and MAPK8 proteins.

#### 3.5.7 MMPBSA calculation

**FIGURE 18:**
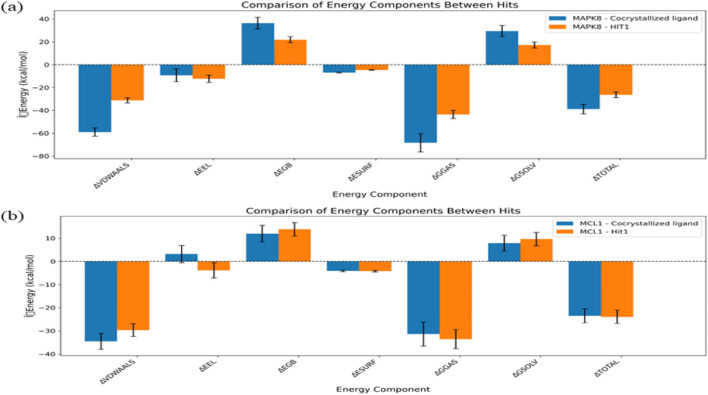
MMPBSA calculation Hit1 and co-crystallized ligand in the binding pocket of **(a)** MAPK8, **(b)** MCL-1.

To confirm the stability of the selected protein-ligand complexes: MAPK8-Hit1, MAPK8-Co-crystallized ligand, MCL-1-Hit1, MCL-1-Co-crystallized ligand, NLRP3-Hit1, NLRP3-Co-crystallized ligand, TNF- α -Hit1, and TNF- α -Co-crystallized ligand complexes, we conducted the MMPBSA calculations for the last 1,000 frames of each trajectory using the gmx_MMPBSA tool. As the outcome of the study, we identified the total binding energy, which is the combination of different other energies such as Vander Waals contribution (ΔVDWAALS), electrostatic energy (ΔEEL), Polar solvation-free energy (ΔEGB), non-polar solvation-free energy (ΔESURF), Gas face free energy (ΔGGAS), and solvation energy (ΔGSOLV) (Table 2). In the current study, on the MAPK8 target, the total binding energy for MAPK8-Hit1 and MAPK8-Co-crystallized ligand was found to be −26.33 ± 2.43 kcal/mol and −38.87 ± 4.21 kcal/mol, respectively. This clearly depicted the greater stability of the co-crystallized ligand than the Hit1 molecule in the binding pocket of the MAPK8 protein (Figure 18a).

**FIGURE 19:**
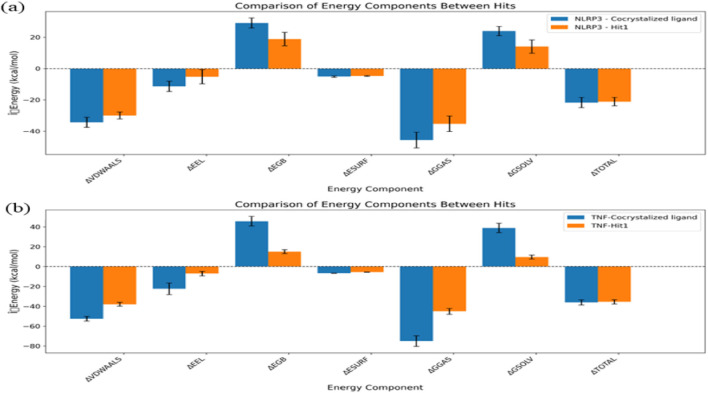
MMPBSA calculation Hit1 and co-crystallized ligand in the binding pocket of **(a)** NLRP3, and **(b)** TNF- α proteins.

In contrast, on the MCL-1 target, the total binding energies were found close to each other, and they were −23.92 ± 2.90 kcal/mol and −23.49 ± 3.05 kcal/mol for MCL-1-Hit1 and MCL-1-Co-crystallized ligand, respectively. Although the total binding energies for the two complexes were very close to each other, when we relate it to the previous analysis, the superior nature of the Hit1 over the co-crystallized ligand could be easily assumed (Figure 18b). Similarly, the total binding energies for NLRP3-Hit1 and NLRP3-Co-crystallized ligand were estimated as −21.14 ± 2.67 kcal/mol and −21.72 ± 3.25 kcal/mol, respectively. This again confirms the greater stability of Hit1 than the co-crystallized ligand in the binding pocket of the NLRP3 target (Figure 19a).

Lastly, on the TNF- α target, the observed total binding energies of TNF- α _Hit1 and TNF- α -Co-crystallized ligand were −35.52 ± 2.20 kcal/mol and −36.15 ± 2.62 kcal/mol, respectively. The comparison with previous analysis for the TMF target did not align; however, close total binding energies could be assumed as similar behavior of Hit1 and the co-crystallized ligand in the binding pocket of TNF protein (Figure 19b).

The protein-ligand interactions were stable over the entire MDS as evidenced by consistently low RMSD, RMSF, RG, SASA, hydrogen bonding and MM-PBSA profiles throughout the simulations; however, these results should be interpreted as showing computationally stable binding in a simulated environment rather than as conclusive evidence of biological activity; hence, additional experiments will be needed to validate these results.

## 4 Discussion

This study utilized an integrated network pharmacology and molecular modelling strategy to examine the potential molecular interactions of phytochemicals from *Arctium lappa* against hepatotoxicity and pancreatitis induced by VPA. HR-LCMS-QTOF analyses identified 111 metabolites, of which 22 compounds exhibited favourable ADMET and toxicity profiles, suggesting potential drug-like characteristics. Previous studies have indicated that phytochemicals possessing multi-target pharmacological properties may interact with multiple biological pathways involved in complex pathological conditions, including drug-induced organ toxicity.

Network pharmacology analysis identified 18 common targets associated with both phytochemical related targets and VPA-induced toxicity pathways. Key hub proteins including TNF-α, MAPK8, NLRP3, MCL-1, HSP90AA1, and PTGS1 were associated with inflammatory signaling, oxidative stress, and apoptotic processes (Thomas et al., 2010; Li et al., 2018). Previous studies have reported that TNF-α, and MAPK8 signaling are involved in hepatic inflammation and pancreatic injury following VPA exposure, while NLRP3 inflammasome activation and pancreatic injury following VPA exposure, while NLRP3 inflammasome activation contributes to caspases-1 mediated inflammatory response and apoptosis (Cargnello and Roux, 2011; Kelley et al., 2019; Yu et al., 2020). The identification of these targets in the present analysis suggests their potential relevance to the molecular mechanisms underlying VPA-induced toxicity.

Functional enrichment analysis highlighted apoptosis, IL-17 signaling, cytochrome P450-mediated metabolism, and NOD-like receptor signaling as pathways potentially associated with toxicity progression. These observations are consistent with previous reports indicating that oxidative stress, altered xenobiotic metabolism, and inflammatory cytokines signalling contribute to VPA-induced liver and pancreatic injury. Furthermore, the enrichment of NF-κB and MAPK related pathways suggests possible interactions among inflammatory and apoptotic signaling network involved in toxicity development (Cargnello and Roux, 2011; Kelley et al., 2019).

Molecular docking analyses demonstrated favourable predicted binding affinities between selected phytochemicals and targets including MAPK8, MCL-1, NLRP3, and TNF-α. Among the evaluated compounds 4-Hydroxy-3-methoxy-2,10-bisaboladien-9-one exhibited comparatively strong predicted interactions throughout hydrogen bonding, hydrophobic contacts, and π-interactions. Molecular dynamics simulations over 200 ns supported the stability of these predicted ligand-target complexes, particularly with MCL-1 and NLRP3, as indicated by RMSD, RMSF, SASA, PCA and free energy landscape analyses. Similar computational and experimental studies have suggested that bisabolene-type sesquiterpenoids may interact with signalling pathways related to inflammation, oxidative stress, and inflammasome activation.

The present study is notable for identifying common toxicity-associated hub targets that link VPA-induced hepatotoxicity and pancreatitis. Network pharmacology study identified NLRP3, MAPK8, TNF-α, and MCL-1 as important regulatory nodes for inflammation, oxidative stress, mitochondrial dysfunction, and apoptosis. NLRP3 plays a key role in activating inflammasomes and releasing pro-inflammatory cytokines. TNF-α and MAPK8 are linked to inflammatory and stress-related signaling pathways. MCL-1 has emerged as a key regulator of mitochondrial integrity and cell survival. The simultaneous identification of these targets in both the hepatic and pancreatic damage networks supports the presence of shared molecular pathways driving VPA-induced organ toxicity, highlighting these proteins as promising targets for future mechanistic and therapeutic research.

The integration of network pharmacology, molecular docking and molecular dynamics simulations identified several phytochemicals from *A. lappa*, particularly 4-Hydroxy-3-methoxy-2,10-bisaboladien-9-one, as compound of interest based on their predicted interactions with targets associated with oxidative stress, inflammation, and apoptosis. However, these findings are derived exclusively from computational analyses and should therefore be considered predictive and hypothesis-generating. Furthermore, *in-vitro* and *in-vivo* are necessary to validate the proposed molecular interactions, biological activities, pharmacokinetic properties, and potential relevance of these compounds in VPA-induced toxicity.

### 4.1 Limitation of the study

The study is based entirely on computational approaches, including network pharmacology, molecular docking, molecular dynamics simulations, and ADMET predictions. Therefore, the findings are dependent on the accuracy of available databases and predictive algorithms. Additionally, the identified targets and pathways represent predictive associations and may not fully reflect *in-vivo* biological complexity. Although stable ligand-target interactions were observed *in silico*, experimental validation was not performed. Consequently, the result should be considered hypothesis-generating and require further *in-vitro* and *in-vivo* studies for conformation.

## 5 Conclusion

This study employed an integrated network pharmacology and molecular modelling framework to investigate the potential system-level mechanisms associated with VPA-induced hepatotoxicity and pancreatitis. Phytochemical profiling of *A. lappa* identified several bioactive compounds with favourable predicted ADMET characteristics. Network analysis highlighted key molecular targets, including MAPK8, NLRP3, TNF-α and MCL-1, which are associated with inflammatory responses, oxidative stress, and apoptotic processes. Molecular docking and molecular dynamics simulations suggested stable interactions between selected phytochemicals, particularly 4-Hydroxy-3-methoxy-2,10-bisaboladien-9-one, and targets such as MCL-1 and NLRP3, indicating their possible involvement in pathways relevant to VPA-induced toxicity. As these findings are derived exclusively from computational analyses, they should be regarded as predictive and hypothesis-generating rather than experimentally validated evidence. Nevertheless, the study provides mechanistic insight into the molecular networks potentially involved in VPA-induced organ injury and identifies candidate phytochemical and targets that warrant further investigation through *in-vitro* and *in-vivo* experimental studies.

## Data Availability

The raw data has been deposited to the GitHub repository: https://github.com/mukulshyam/Valproic_acid_induced_Toxicity.
