# Supplementary material for: Network pharmacology and molecular modelling analysis of Arctium lappa phytochemicals in valproic acid–induced hepatotoxicity and pancreatitis
Source: Front Bioinform. 2026 Jul 20;6:1870490. doi: 10.3389/fbinf.2026.1870490 (PMC13429713; doi:10.3389/fbinf.2026.1870490)
Supplement: Supplementary file 1 [file Supplementaryfile1.docx]

**Network Pharmacology and Molecular Modelling Analysis of *Arctium lappa* Phytochemicals in Valproic Acid–Induced Hepatotoxicity and Pancreatitis**

Mukul Shyam and Sabina Evan Prince^*^

School of Bio Sciences and Technology, VIT University, Vellore, India

Supplementary file

Figure S1- Chromatogram in ESI_Positive and ESI_Negative


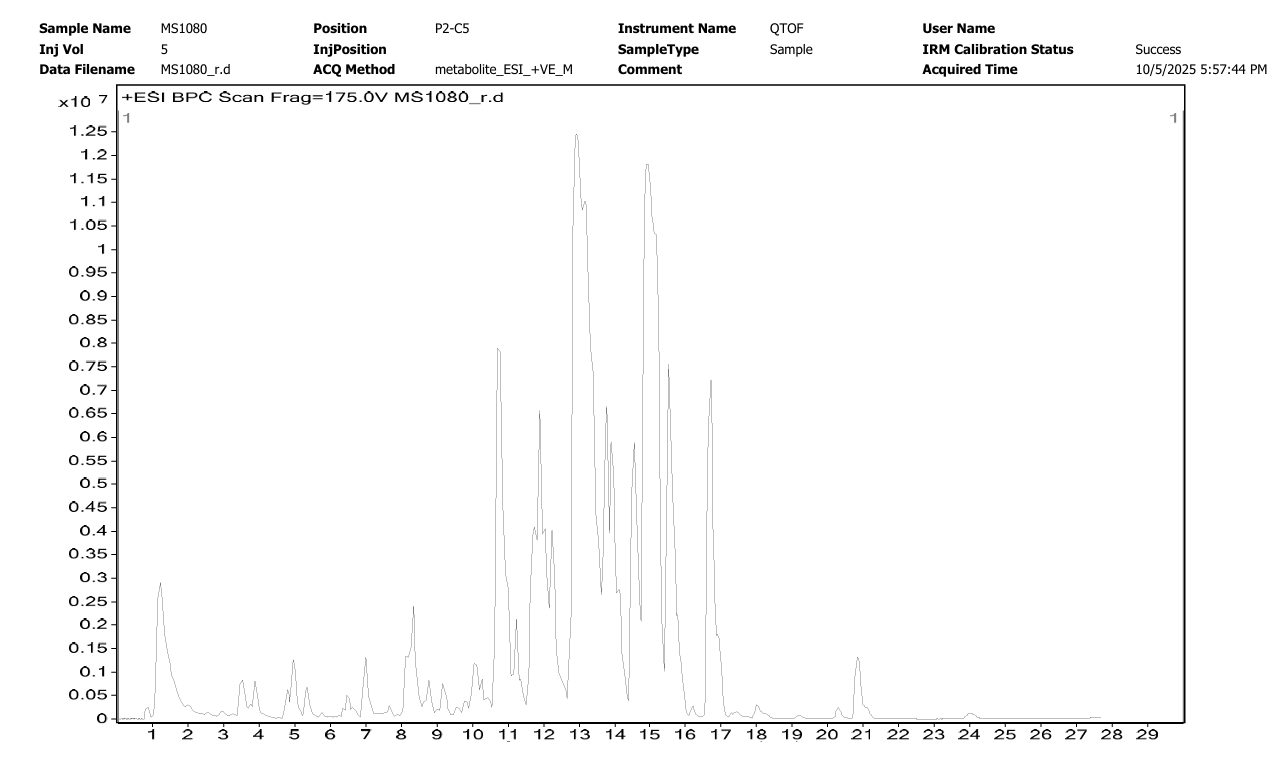


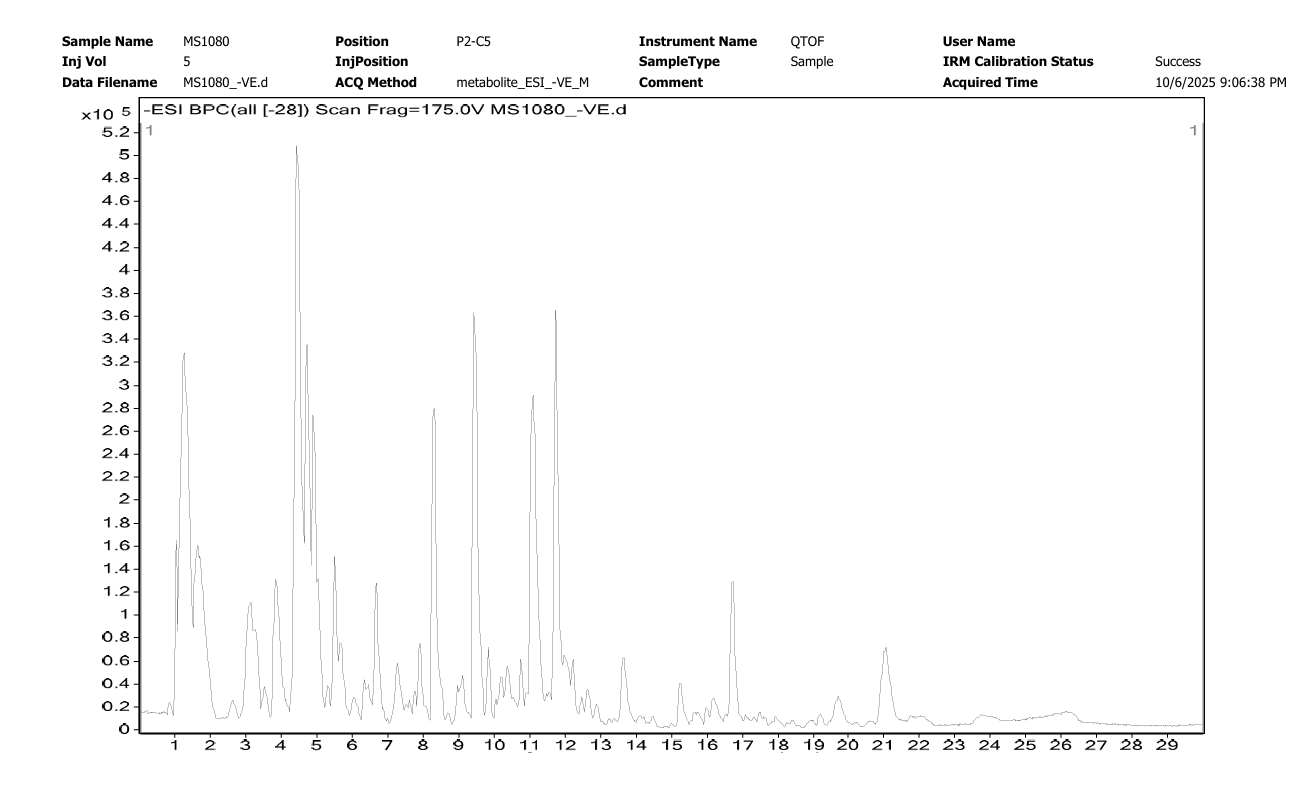


Table S1

| Target symbol | Name of target | PDB ID | Resolution | Methos | Key Residue in pocket | Grid size |
| --- | --- | --- | --- | --- | --- | --- |
| MAPK8 | Mitogen-activated protein kinase 8 | 4QTD | 1.50 Å | X-ray | ILE32, GLY33, SER34, GLY35, GLN37, GLY38, VAL40, VAL52, ALA53, ILE54, LYS55, ILE86, MET108, GLU109, LEU110, MET111, ASP112, ALA113, ASN114, CYS116, GLN117, VAL118, GLN120, LYS153, SER155, ASN156, VAL158, VAL159, LEU168, ASP169 | center_x = 20.332  center_y = 9.780  center_z = 30.015  size_x = 21.75  size_y = 24  size_z = 28.5 |
| MCL-1 | Induced myeloid leukemia cell differentiation protein Mcl-1 | 5FC4 | 1.50 Å | X-ray | HIS224, THR226, ALA227, PHE228, GLN229, GLY230, MET231, LEU232, LEU235, LEU246, VAL249, MET250, VAL253, VAL258, ASN260, GLY262, ARG263, ILE264, VAL265, THR266, LEU267, ILE268, PHE270, GLY271, VAL274, LEU290, ILE294 | center_x = 4.182  center_y = 4.795  center_z = 13.063  size_x = 25.5  size_y = 21  size_z = 27 |
| NLRP3 | NACHT, LRR and PYD domains-containing protein 3 | 9GU4 | 2.70 Å | X-ray | GLY226, ALA227, ALA228, GLY229, ILE230, ARG351, PRO352, VAL353, MET408, PHE410, ILE411, LEU413, VAL414, ILE417, THR439, VAL442, TYR443, HIS522, THR524, TYR565, ILE574, PHE575, ARG578, PHE579, GLN624, SER626, LEU628, GLU629, LEU630, TYR632, GLU636, THR659, MET661, ASP662, VAL665 | center_x = 14.297  center_y = 32.142  center_z = -6.926  size_x = 27.75  size_y = 24  size_z = 24.75 |
| PTGS1 | Prostaglandin G/H synthase 1 | 6Y3C | 3.36 Å | X-ray | CYS36, TYR39, PRO40, CYS41, GLN42, HIS43, GLN44, GLY45, ILE46, CYS47, TYR130, ASP135, ILE151, LEU152, PRO153, SER154, VAL155, PRO156, GLN461, GLU465, LYS468, ARG469 | center_x = -15.704  center_y = -58.494  center_z = 0.990  size_x = 19.5  size_y = 24  size_z = 17.25 |
| TNF | Tumor necrosis factor | 9OJO | 1.36 Å | X-ray | LYS11: A, LEU57: A, TYR59: A, TYR119: A, VAL123: A, ILE155: A, ALA156: A, LEU157: A, LEU57: B, TYR59: B, SER60: B, GLN61: B, TYR119: B, LEU120: B, GLY121: B, GLY122: B, TYR151: B, ILE155: B, LEU157: B, LEU57: C, ILE58: C, TYR59: C, TYR119: C, GLY121: C, GLY122: C, VAL123: C, ILE155: C, LEU157: C | center_x = -13.532  center_y = -1.245  center_z = 20.044  size_x = 24  size_y = 21  size_z = 24 |

Table S2

| Compound_ID | Common name | MAPK8 | MCL-1 | NLRP3 | PTGS1 | TNF |
| --- | --- | --- | --- | --- | --- | --- |
| CID: 10883 | Ethyl levulinate | -4.7 | -4.2 | -4.7 | -5.2 | -5.4 |
| CID: 11902 | Methyl 2-furoate | -4.5 | -3.9 | -5.2 | -5 | -5.2 |
| CID: 11954063 | 9,10-Dihydroxy-12,13-epoxyoctadecanoate | -6.9 | -5.4 | -6.1 | -6.4 | -8.0 |
| CID: 120773 | Tecostanine | -6.2 | -5.0 | -5.6 | -6.0 | -7.1 |
| CID: 131751504 (Hit1) | 4-Hydroxy-3-methoxy-2,10-bisaboladien-9-one | **-7.6** | **-6.6** | **-7.5** | -7.0 | **-8.7** |
| CID: 14256 | Dodecanamide | -5.8 | -5.1 | -5.2 | -5.6 | -6.2 |
| CID: 20392 | Monomethyl phthalate | -6.0 | -4.8 | -5.8 | -6.1 | -7.1 |
| CID: 213060 | 2-Phenyl-1,3-propanediol monocarbamate | -6.4 | -5.5 | -6.0 | -6.6 | -7.9 |
| CID: 222656 | L-Malic Acid | -4.7 | -3.5 | -5.8 | -5.1 | -4.6 |
| CID: 22394751 | (Z)-3-(1-Formyl-1-propenyl)pentanedioic acid | -6.1 | -5.1 | -5.5 | -6.1 | -6.5 |
| CID: 2576 | Carisoprodol | -6.3 | -5.0 | -5.5 | -6.5 | -7.5 |
| CID: 3446 | Gabapentin | -5.5 | -4.8 | -5.7 | -6.1 | -6.2 |
| CID: 442529 (Hit2) | Fabianine | **-7.6** | -5.5 | -6.2 | **-7.9** | **-8.6** |
| CID: 5281118 | alpha-Licanic acid | -6.6 | -5.7 | -6.0 | -7.2 | -8.2 |
| CID: 533941 | Methyl N-isovalerylglycine | -5.4 | -4.4 | -5.1 | -5.8 | -6.0 |
| CID: 580707 | 2-Butyl-4-ethyl-5-methyl-1,3-oxazole | -5.6 | -5.3 | -5.3 | -5.8 | -6.7 |
| CID: 596894 | Kakuol | -6.6 | -5.8 | -6.5 | -6.7 | -7.0 |
| CID: 66021 | Diacetin | -5.4 | -4.0 | -5.2 | -5.5 | -5.7 |
| CID: 71347088 | 1-(5-Methyl-3-pyridinyl)-1-decanone | -6.5 | -6.3 | -6.3 | -6.5 | -7.8 |
| CID: 86025817 | 4-Ethyl-2-hexyl-5-methyloxazole | -6.2 | -5.6 | -5.6 | -6.0 | -7.2 |
| Co-crystallized ligand | Reference | **-12.1** | **-8.3** | **-10.7** | **-** | **-12.8** |
